# Effects of an unusual poison identify a lifespan role for Topoisomerase 2 in *Saccharomyces cerevisiae*

**DOI:** 10.18632/aging.101114

**Published:** 2017-01-05

**Authors:** Gregory Tombline, Jonathan I. Millen, Bogdan Polevoda, Matan Rapaport, Bonnie Baxter, Michael Van Meter, Matthew Gilbertson, Joe Madrey, Gary A. Piazza, Lynn Rasmussen, Krister Wennerberg, E. Lucile White, John L. Nitiss, David S. Goldfarb

**Affiliations:** ^1^ Biology Department, University of Rochester, Rochester, NY 14627, USA; ^2^ Drug Discovery Division, Southern Research Institute, Birmingham AL, 35205, USA; ^3^ Department of Biopharmaceutical Sciences, UIC College of Pharmacy at Rockford, Rockford, IL 61107, USA

**Keywords:** replicative lifespan, aging, topoisomerase 2 poison, DNA damage, antagonistic pleiotropy

## Abstract

A progressive loss of genome maintenance has been implicated as both a cause and consequence of aging. Here we present evidence supporting the hypothesis that an age-associated decay in genome maintenance promotes aging in *Saccharomyces cerevisiae* (yeast) due to an inability to sense or repair DNA damage by topoisomerase 2 (yTop2). We describe the characterization of LS1, identified in a high throughput screen for small molecules that shorten the replicative lifespan of yeast. LS1 accelerates aging without affecting proliferative growth or viability. Genetic and biochemical criteria reveal LS1 to be a weak Top2 poison. Top2 poisons induce the accumulation of covalent Top2-linked DNA double strand breaks that, if left unrepaired, lead to genome instability and death. LS1 is toxic to cells deficient in homologous recombination, suggesting that the damage it induces is normally mitigated by genome maintenance systems. The essential roles of yTop2 in proliferating cells may come with a fitness trade-off in older cells that are less able to sense or repair yTop2-mediated DNA damage. Consistent with this idea, cells live longer when yTop2 expression levels are reduced. These results identify intrinsic yTop2-mediated DNA damage as a potentially manageable cause of aging.

## INTRODUCTION

The longevity of an organism is determined by a confounding combination of genes and environment. Lifespan assays in model organisms have identified numerous longevity genes—those that when mutated or when over- or underexpressed affect mean and/or maximum lifespan—with primary roles in many cell processes, including genome maintenance, metabolism, mitochondrial function, and oxidative stress [1, 2]. The lifespan of *Saccharomyces cerevisiae* (yeast) is measured using chronological or replicative models, both of which respond similarly to environmental interventions such as caloric restriction and oxidative stress [3]. While there is no correlation between the chronological and replicative lifespans of natural yeast isolates [4], the two methods have yielded overlapping sets of longevity genes, and both have identified longevity pathways or genes with relevance to mammalian aging. The standard method for quantifying replicative lifespan (RLS) is slow and tedious because it requires the manual microdissection of daughter cells from their mothers, but see [5, 6]. In this study we describe improvements to the high throughput capable Death of Daughters (DeaD) RLS proxy assay [7], and employ it in a screen for small molecules that shorten RLS without affecting proliferation or viability.

Here we characterize LS1, a RLS shortening molecule that acts by poisoning yeast topoisomerase 2 (yTop2). yTop2 is an essential enzyme that generates transient double strand breaks (DSBs) in order to relieve positive and negative DNA supercoils during replication, transcription and DNA repair, and to separate tangled (concatenated) chromosomes prior to mitosis [8]. Top2 poisons stabilize Top2-DNA covalent complexes (Top2ccs) in which the 5′-phosphate ends of the double strand break are linked to the enzyme via phosphotyrosine ester bonds. If left unrepaired Top2ccs disrupt transcription and replication and lead to genome instability, senescence and cell death [9, 10]. Top2 poisons include widely used chemotherapeutic drugs, but are also found in foods and in the environment [11]. DNA abasic sites, alkylated bases, and UV-induced lesions are also capable of stabilizing Top2-DNA adducts [11]. This is relevant to the potential role of Top2 in aging because all of these base modifications and lesions accumulate in aging cells and tissues [12]. Genetic programs that recognize and repair Top2ccs, including specific tyrosyl phosphodiesterases and nucleases [13–15] and DNA repair systems [16, 17], support the notion that Top2ccs are a normal fact of life. Chemotherapeutic Top2 poisons kill dividing (cancer) cells by overloading these remediation mechanisms with large numbers of Top2ccs.

The simplest explanation for our results is that LS1 is selectively toxic to aging cells that have a diminished capacity to repair Top2ccs. In support of this hypothesis we show that reducing *TOP2* expression is sufficient to extend RLS. Previous evidence in support of the DNA damage theory of aging is based on observations and experiments that link DNA damage to reductions in longevity. Our results show that native levels of DNA damage by yTop2 are a direct cause of aging.

## RESULTS

### Death of Daughters (DeaD) assay recapitulates aspects of replicative aging

The DeaD assay is a high throughput proxy for the standard RLS microdissection assay, which is slow and labor intensive [7]. The DeaD assay is based on the W303R derived strain K6001 that was engineered to study mother cell specific mating type switching [18, 19]. As described in the Supplementary Data we reconstructed and greatly improved the performance of the original DeaD strain (Supplemental Figs. S1, S2 & S3). DeaD strains contain two chromosomal copies of the essential *CDC6* gene, one under the control of the inducible *GAL1* promoter and the other under the control of the mother-specific *HO* promoter. In galactose-containing medium, both mothers and daughters express *CDC6*, predominately from the *GAL1* promoter, and divide exponentially. In glucose-containing medium, daughter division is strongly inhibited due to the lack of expression of *CDC6* from either promoter, and the growth rate and saturation point of the culture becomes limited by the reproductive capacity of the mother cells rather than nutrient limitation.

The DeaD assay recapitulates lifespan shortening and extension associated with under- and over-expression of *SIR2* (Fig. S3). The DeaD assay also recapitulates lifespan shortening by a number of gene deletions previously shown by microdissection assay to shorten RLS, including *sir2Δ*, *sgs1Δ*, *rad9Δ*, *rad51Δ*, *rad52Δ*, *phb1Δ*, and *isw1Δ* (Fig. S4), but does not show RLS extension in many strains containing gene deletions known to extend RLS (including *tor1Δ* and *hxk2Δ*). Therefore, in this report we focused on the demonstrated utility of the DeaD assay to report RLS shortening.

### High throughput screen for small molecules that shorten replicative lifespan

Small molecules that reduce DeaD cell growth under restrictive conditions, but minimally affect growth under permissive condition, are candidate probes that target longevity factors. An essential criterion for meaningful RLS shortening is a lack of toxicity at doses where statistically significant RLS shortening is observed. It is not interesting to shorten “apparent” RLS simply by making cells sick.

The DeaD assay was used to screen a 138,758 compound NIH small molecule library for those that reduced growth in glucose (restrictive) without significantly affecting growth in galactose (permissive) (see PubChem Assay #AID 804). 759 active compounds were reassessed under both restrictive and permissive conditions in a 10-point 2-fold dilution series ranging from 0.098 - 50 μM (see PubChem Assay #AID 849). The top 44 DeaD lifespan-shortening compounds were ranked according to their ratios of restrictive growth/permissive growth (Supplementary Methods). Here we describe studies on the biological activities and mechanism of action of LS1 (6H-Indolo [2,3-b] quinoxaline: SID 4264584).

### LS1 is a potent lifespan shortener

**Figure 1:**
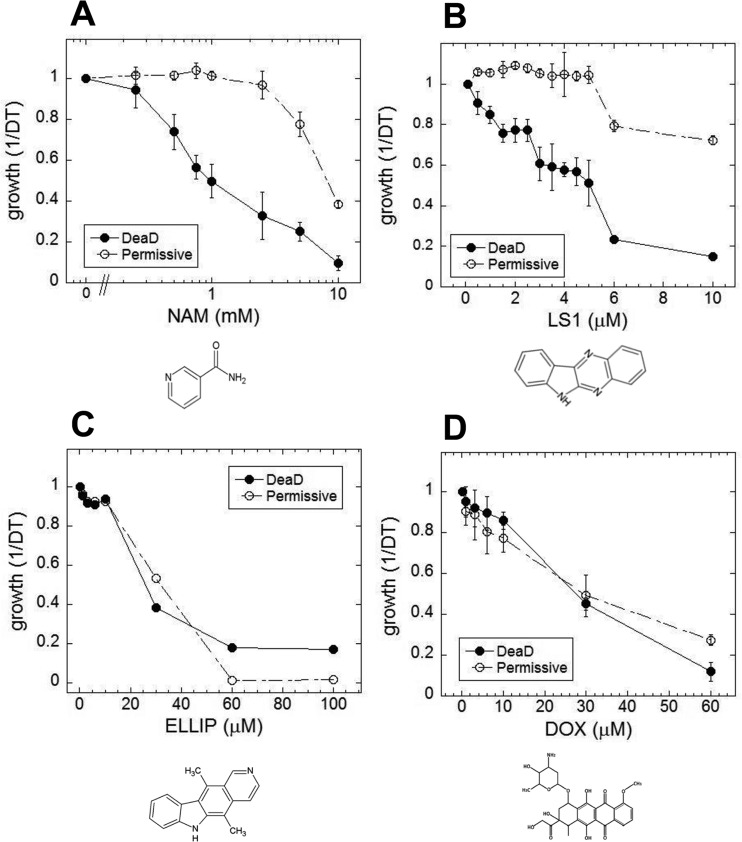
Small molecule effects on DEAD lifespans Growth rates of DEAD strain (BB579) in the presence of increasing concentrations of nicotinamide **(**NAM) (**A**), LS1 (**B**), ellipticine (ELLIP) (**C**) and doxorubicin (DOX) (**D**) under nonpermissive (●) and permissive (○) conditions.

In yeast, nicotinamide (NAM) shortens RLS largely due to the compound's activity as a feedback inhibitor of Sir2, though it inhibits other yeast sirtuins that affect lifespan and also prevents lifespan extension by caloric restriction by a sirtuin-independent mechanism [20–22]. As shown in Fig. 1A, NAM shortens DeaD lifespan at concentrations (IC_50_ = 1 mM) where it has no discernable effect on permissive growth. Similar to NAM, LS1 also exhibits dose-dependent DeaD lifespan shortening (DeaD IC_50_ = 5 μM) at concentrations where permissive growth is unaffected (Fig. 1B), though it is ∼200-fold more potent than NAM. The DeaD assay RLS shortening activity of 1 μM LS1 was confirmed by standard microdissection assay (Fig. 2).

**Figure 2:**
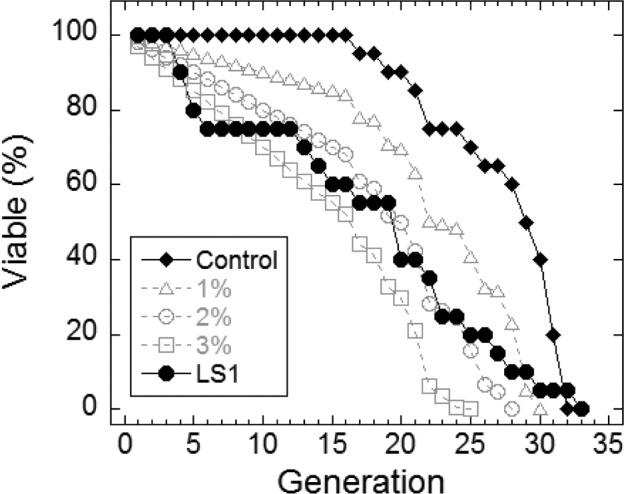
LS1 shortens replicative lifespan by standard microdissection assay Microdissection in the presence and absence of 1μM LS1 was performed as described in Materials and Methods. LS1 treated (●) FY839 cells had an average lifespan of 16.1 generations, a 41.5% drop from the untreated sample (○). Graph shows combined data from two independent FY839 colonies. Superimposed over the experimental data are modeled data showing the effect on the lifespan of the untreated sample of adding 1% (Δ), 2% (○) and 3% (□) non-age-related cell death/generation.

### LS1 acts independently from the role of Sir2 in ERC accumulation

The RLS of yeast is normally limited by the age-dependent accumulation of extrachromosomal rDNA circles (ERCs) [23]. ERC formation requires Fob1 and is suppressed by Sir2 activity [24, 25]. Thus *fob1Δ* cells are resistant to the lifespan shortening effects of *SIR2* deletion and NAM [24]. To test whether LS1 acts in this pathway, for example, by directly or indirectly inhibiting Sir2, we assessed the effects of LS1 in *fob1Δ* and *sir2Δ* strains. As shown in Fig. 3A, LS1 effectively reduced DeaD lifespans in both *fob1Δ* and *sir2Δ* strains. In contrast, NAM, which acts predominately by inhibiting Sir2, shows significantly less DeaD lifespan shortening activity in *fob1Δ* and *sir2Δ* strains (Fig. 3B). The modest lifespan reduction by NAM in *fob1Δ* and *sir2Δ* strains may be due to its' sirtuin-independent RLS-shortening activities [22]. We conclude that LS1 shortens RLS by an ERC- and Sir2-independent mechanism.

**Figure 2:**
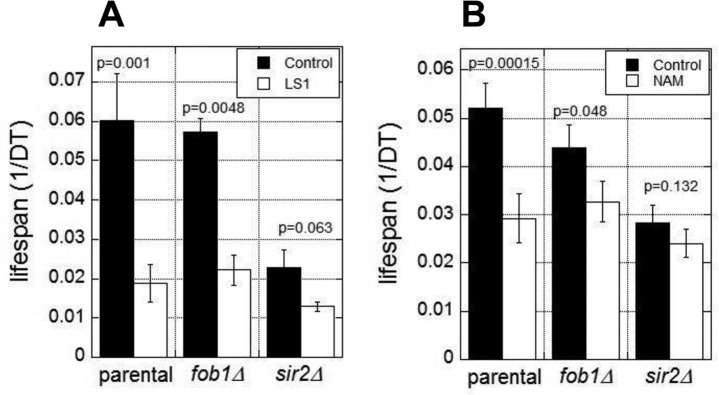
Lifespan shortening activity of LS1 is independent of 2μ circles and Sir2 DEAD assays were performed in the presence and absence of 5μM LS1 (**A**) or 1mM NAM (**B**) in parental (BB579), *fob1*Δ, or *sir2*Δ strains as described in Materials and Methods. Data represent the average of three biological replicates. Error bars represent standard deviation.

**Figure 4:**
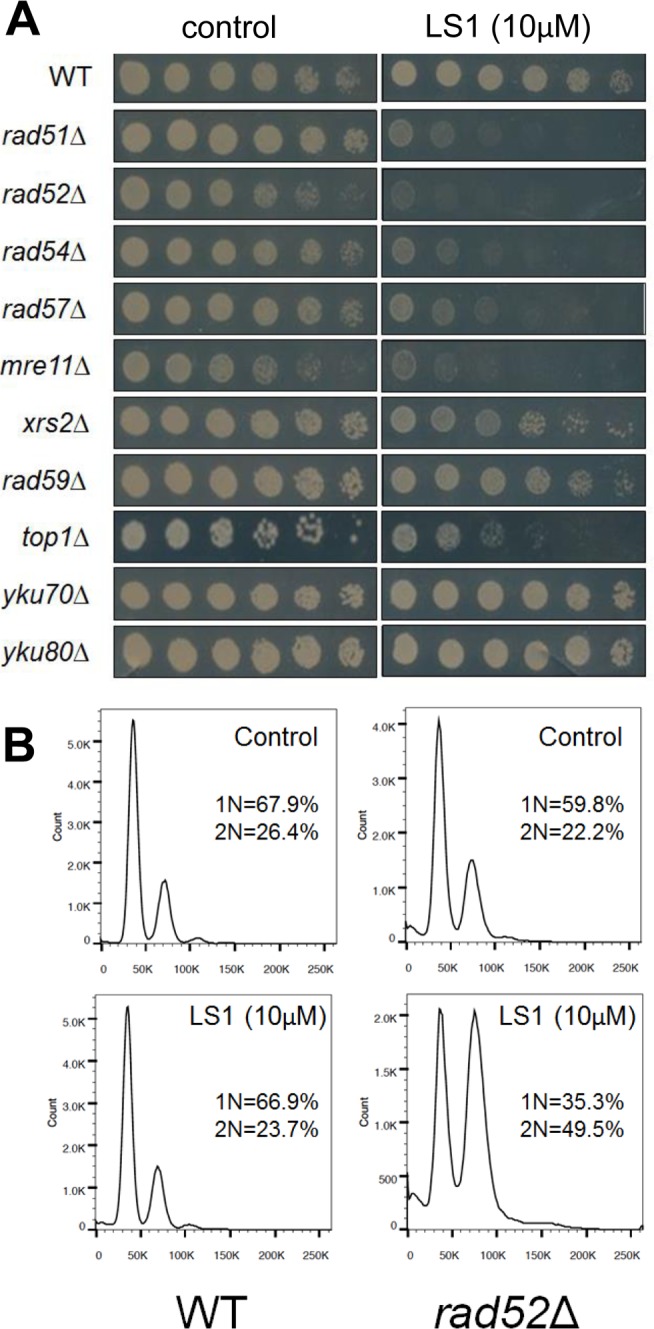

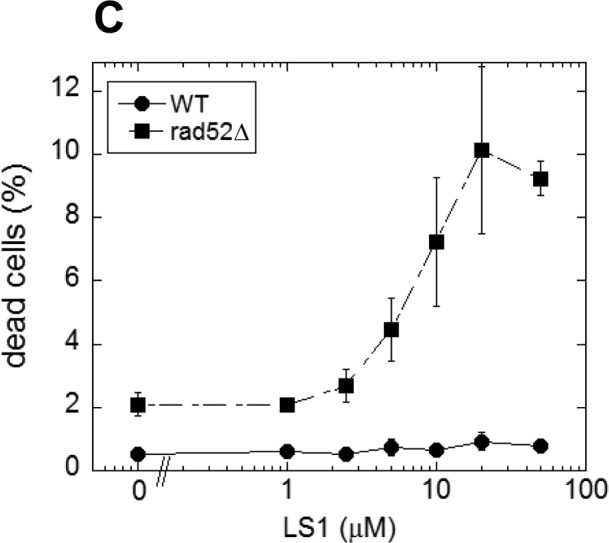
Synthetic genetic interactions of LS1 with *RAD52* epistasis group deletions A comprehensive screen of the non-essential deletion collection revealed synthetic growth defects of LS1 together with deletions of genes required for homologous recombination. (**A**) Three-fold dilutions (starting at 0.1 OD_600_) of log phase growing cultures of parental (BY4741) and deletion strains were plated at 30°C for 48 h onto SCD agar containing 0.1% DMSO vehicle (control) without or with 10μM LS1. (**B**) DNA content by flow cytometry of LS1 treated parental BY4741 and *rad52*Δ cells. Log phase liquid cultures were treated with 0.1% DMSO vehicle (control) without or with 10μM LS1 for 6h followed by fixation with ethanol and propidium iodide staining as described in Materials and Methods. (**C**) Quantitative effect of increasing concentrations of LS1 on cell viability in log cultures of *RAD52* and *rad52*Δ cells. Quantitation of cell viability is described in Materials and Methods.

### LS1 is toxic in cells lacking homologous recombination

To gain insight into the mechanism of action of LS1 we performed a genome-wide chemical-genetic screen for deletions of nonessential genes that caused slow growth in the presence of LS1. This screen identified a set of genes belonging to the *RAD52* epistasis group required for homologous recombination. Specifically, LS1 caused a slow growth phenotype in *rad51Δ*, *rad52Δ*, *rad54Δ*, *rad57Δ*, and *mre11Δ* cells. LS1 had no vegetative growth effect on y*ku70Δ* or y*ku80*Δ cells that are defective in DSB repair by nonhomologous end joining (NHEJ) (Fig 4A). Thus HR, but not NHEJ, is necessary for resistance to LS1. FACS analysis revealed that LS1-treated *rad52Δ* cells tended to arrest growth at G2/M (Fig. 4B). Parental cells treated with LS1 did not show a cell cycle phenotype. The growth defect of *top1Δ* cells in the presence of LS1 was of interest, since the only class of DNA damaging agents that have been reported to result in enhanced growth defects in *top1Δ* mutants are Top2 poisons [26]. *top1Δ* mutants are hypersensitive to Top2 poisons because these cells rely on Top2 for all topoisomerase functions that are normally shared by Top1 and Top2 [9, 10, 27]. Thus the sensitivity of *top1Δ* cells is fully consistent with LS1 being an yTop2 poison.

The synthetic growth defect of LS1 in *rad52Δ* cells is quantified in Fig. 4C and shows that the slow growth phenotype of *RAD52*^+^ cells at very high LS1 concentrations is not due to cell death (Fig. 1B). Interestingly, these data show that in the absence of LS1 *rad52Δ* log cultures contain a steady state level of ∼2% dead cells compared with less than 0.5% in *RAD52*^+^ cultures (Fig. 4C). Although a death rate of ∼2% per generation would be undetectable by standard methods used to quantify growth, these levels are amplified in RLS studies. Adding 1%, 2%, or 3% non-age-associated cell death/generation reduces the mean RLS of parental cells by 19%, 31% and 45%, respectively (Fig. 2). Therefore, the observed ∼30% reduction in mean RLS caused by 1 μM LS1 could be explained if LS1 killed ∼2% of cells/generation. In fact, LS1 had an insignificant effect on the numbers of dead cells in exponentially dividing cultures, which carry about 0.5% dead cells/generation, even at concentrations as high as 50 μM (Fig. 4). Steady state levels of age-independent cell death in log phase *rad52Δ* cultures could be responsible for the significant fraction of the published short RLS of this and other purportedly short-lived strains [28]. Numbers of nonviable *rad52Δ* cells increased dramatically during stationary phase (not shown), consistent with the report that *RAD52* is required for full chronological lifespan [29]. We conclude that the shorter RLS of LS1-treated cells is not due to low levels of non-age-related cell death, as may be the case for strains such as *rad52Δ*.

Together, these results suggest that the lack of toxicity of LS1 toward young cells at concentrations where it exhibits strong lifespan shortening activity in old cells could be due to an age-associated decrease in the capacity of older cells to sense and repair LS1-induced DNA damage. It follows that the lifespan shortening activity of LS1 could be explained by an age-dependent decline in DNA repair systems [2, 30]. We hypothesize that LS1 is selectively toxic in older cells that have diminished capacity to efficiently either sense or repair LS1-induced DNA damage.

### LS1 is a TOP2 poison

LS1 is basically the unsubstituted scaffold of ellipticine, a Top2 poison [31, 32]. If LS1 were simply a weak Top2 poison, then other Top2 poisons dosed at subtoxic concentrations might also reduce RLS. Two structurally distinct Top2 poisons, ellipticine and doxorubicin, were tested using the DeaD assay for RLS shortening activity. Ellipticine and doxorubicin shorten apparent DeaD lifespan simply by virtue of their being cytotoxic (Fig. 1C & D).

**Figure 5:**
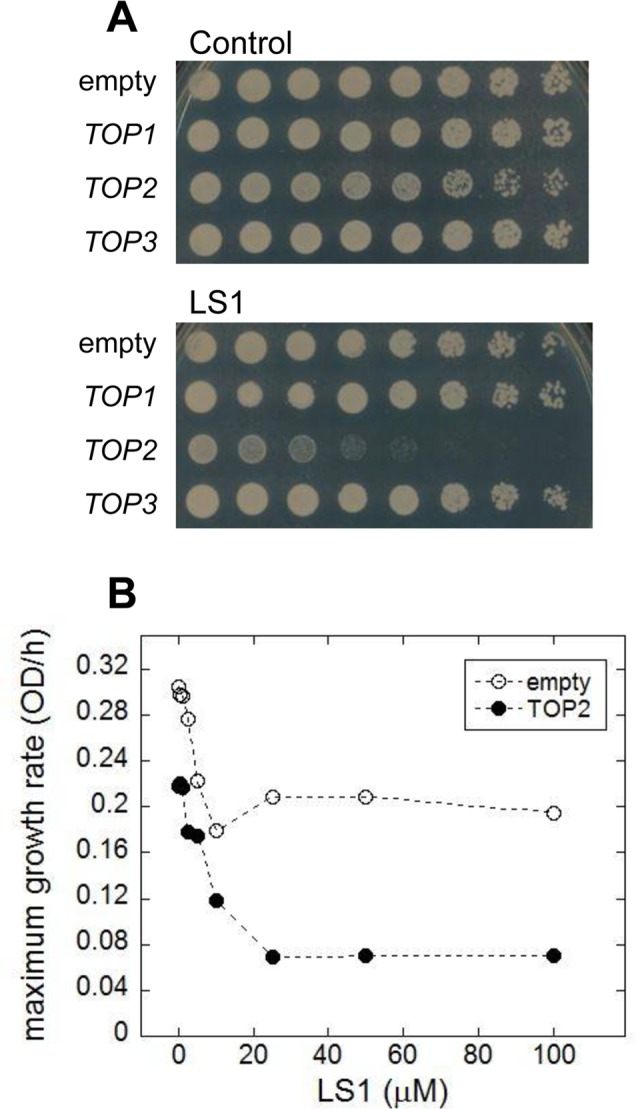
Overexpression of *TOP2* confers hyper-sensitivity to LS1 (**A**) Effect of LS1 on BY4741 transformed with high copy number Yeast Tiling collection plasmids encoding *TOP1*, *TOP2* or *TOP3 (pTOP1, pTOP2, and pTOP3)*. Threefold-dilutions of log phase cells were patched onto YPD medium containing vehicle DMSO (control) or 10μM LS1. Overexpression of *TOP2* was confirmed by quantitative western blot using secondary antibodies conjugated to infrared excitable fluorescent dyes and developed using the *LI-COR* fluorescent imaging system (not shown). (**B**) Quantitative effect of LS1 concentrations on growth of *TOP2* overexpressing strain compared to cells containing the empty vector. Maximum growth rates were calculated from triplicate growth curves generated using a BioScreen C system and BGFit webserver as described in Materials & Methods.

We performed a simple *in vivo* test for yTop2 poisoning by LS1. Because yTop2 participates directly in the formation of cleavage complexes, cells expressing higher levels of yTop2 have the potential to form greater numbers of cleavage complexes and are, therefore, more sensitive to poisons [26]. Consistent with LS1 being a Top2 poison, over-expression of *TOP2*, but not *TOP1* or *TOP3*, increased LS1 toxicity (Fig. 5A). The dose-dependent toxicity of LS1 in cells expressing native or many-fold higher levels of yTop2 is also consistent with a Top2 poisoning mechanism (Fig. 5B). Interestingly, even at high concentrations LS1 did not completely inhibit growth in cells expressing native levels of yTop2. This is interesting because yeast *TOP2* is an essential gene, so strong inhibition should either be lethal or at least cause strong growth defects. It is possible that the cell's repair and remediation pathways cope with a limited number of LS1-induced Top2ccs and, in the steady state, regenerate a sufficient pool of Top2 to perform essential roles. In contrast, doxorubicin and ellipticine completely inhibited growth of cells expressing native *TOP2* levels (Fig. 1C & D). Higher concentrations LS1 had even greater inhibitory effects on the growth of cells overexpressing *TOP2*, indicating that presumptive remediation pathway(s) can be overwhelmed (Fig. 5B).

**Figure 6:**
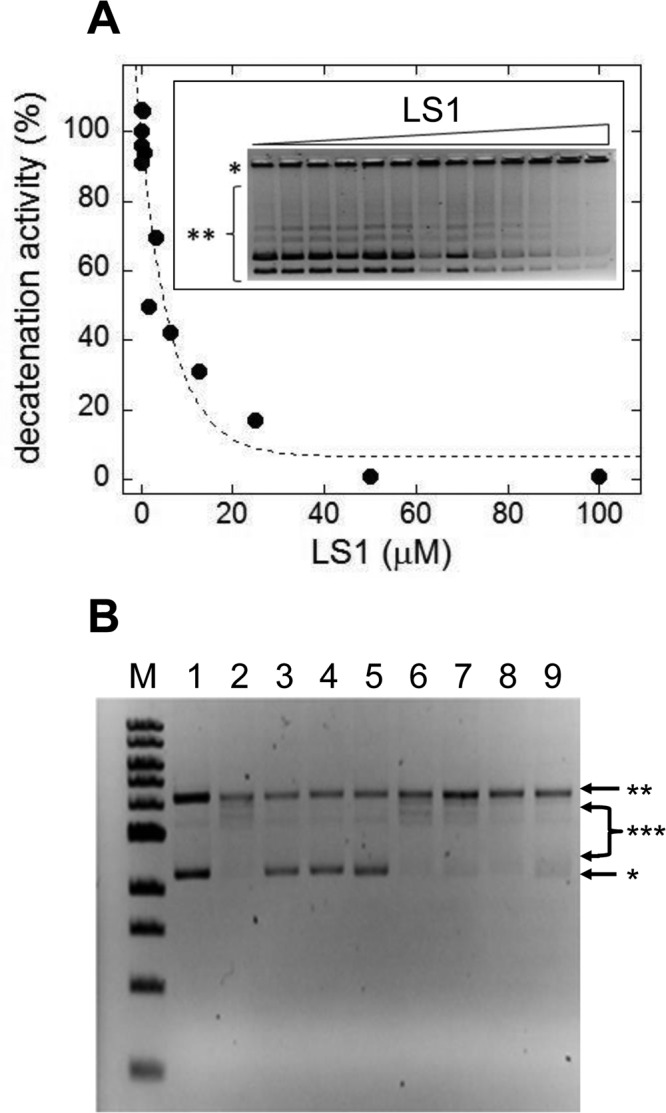
LS1 is a nonintercalating TOP2-α inhibitor (**A**) Effect of LS1 on decatenation of *C. fasciculata* kinetoplastid DNA (kDNA) by purified human TOP2-α. Inset shows a negative image of the ethidium bromide stained agarose gel separating kDNA species. Quantitation of decatenated kDNA was done with GelQuant.NET 1.8.2 software and results were plotted using KaleidaGraph 4.1.0 software. (**B**) A DNA topoisomerase I (Top1) DNA unwinding assay was used to assess the ability of LS1 to intercalate as indicated in Methods. Known Top2 poisons that either intercalate (doxorubicin) or do not (etoposide) were included as controls. An *E. coli*-compatible plasmid (puc18) exhibiting both supercoiled (*) and nicked/relaxed (**) forms was used as the substrate (lane 1). In the absence of an intercalator (lane 2) Top1 converts the plasmid to fully nicked/relaxed (**) or intermediate relaxed forms (***). Intercalation was assessed for DOX (10μM or 50μM; lanes 3-4), ELLIP (100μM; lane 5), 100mM ETOP (lane 6) and LS1 (10μM, 50μM, or 100μM; lanes 7-9).

Based on these *in vivo* results we directly tested LS1 for inhibition of Top2 enzyme activity. Fig. 6A shows that LS1 inhibited kinetoplast DNA decatenation by human Top2α (IC_50_ = 3 μM). Unlike some Top2 poisons, including ellipticine and doxorubicin, LS1 did not detectably intercalate into DNA (Fig. 6B). This finding is consistent with a previous study concluding that LS1 does not bind DNA in contrast to other quinoxaline analogs [33]. A lack of DNA binding or intercalation ability does not by itself explain the lack of toxicity of LS1, since etoposide, another well studied, nonintercalating Top2 poison is cytotoxic.

**Figure 7:**
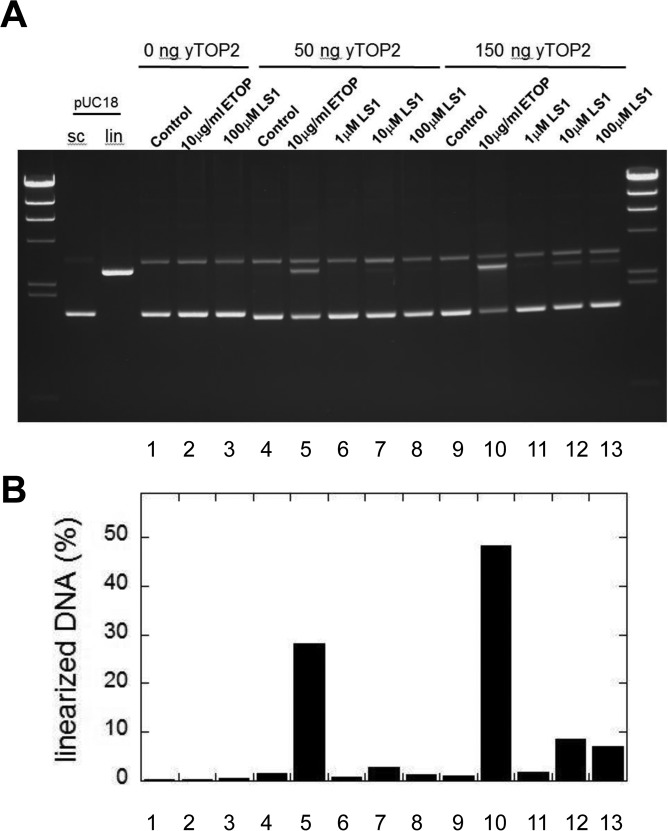
LS1 induces double strand breaks (**A**) A pUC18 plasmid linearization assay was used to determine if LS1 is capable of promoting formation of Top2cc containing double strand breaks. Assays were performed as described in Materials and Methods. (**B**) Percent linearized DNA quantified with GelQuant.NET 1.8.2 software.

Top2 poisons induce the accumulation of DSBs, either by promoting the formation of Top2cc adducts or by slowing their re-ligation, making them aberrantly long-lived. Parenthetically, bisdioxopiperazines such as ICRF-193 promote non-covalent but tightly bound Top2 which are toxic because they interfere with transcription and other DNA metabolic processes; however, because the toxicity of these molecules is not enhanced by mutations in the *RAD52* pathway it is unlikely that DSBs are generated [34, 35]. In support of the conclusion that LS1 is a true Top2 poison, we show that LS1 promotes the *in vitro* formation by yTop2 of DSBs in plasmid DNA (Fig. 7A & B), albeit less efficiently than etoposide.

### LS1 enhances the potency of chemotherapeutic Top2 poisons in yeast and cancer cells

**Figure 8:**
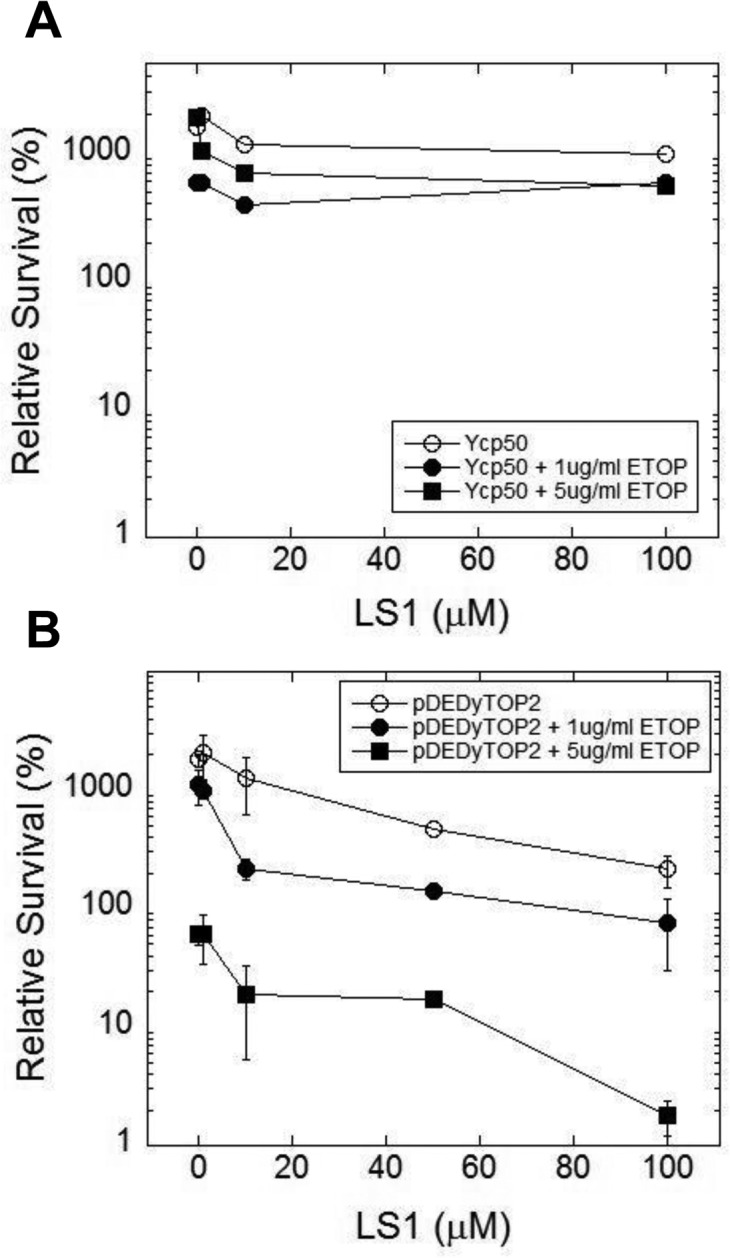
LS1 enhances killing of yeast by etoposide LS1 enhances etoposide (ETOP) cytotoxicity in cells overexpressing *TOP2*. (**A**) Cells expressing native levels of *TOP2* are not sensitive to ETOP in the presence of LS1. Cells contained an empty CEN plasmid Ycp50. (**B**) Overexpression of *TOP2* (YCPpDED1-TOP2) increased sensitivity to ETOP in the presence of LS1. Yeast strain YMM10 [80], which contains gene deletions in several drug efflux pumps, was used to promote ETOP sensitivity.

We hypothesized that LS1 induces the formation of Top2cc adducts that, in the absence of robust homologous recombination, cause cell death by the same mechanism—DNA damage—as classic Top2 poisons. However, unlike other Top2 poisons, LS1 is not toxic at concentrations where it has strong effects on RLS (Fig. 1B). We reasoned that LS1-Top2ccs might be more prone to lethal poisoning by a second more toxic poison. Thus LS1 could at least transiently target Top2 to DNA, effectively increasing the target population for attack by lethal Top2 poisons. As a test we quantified 0, 1 & 5 μM etoposide-induced cell death in the presence and absence of 0-100 μM LS1 in yeast cells expressing native or overexpressed levels of *TOP2*. In cells expressing native levels of Top2, etoposide exhibited little toxicity with or without LS1 (Fig. 8A). As expected from a Top2 poison, etoposide exhibited a strong dose-dependent toxicity in cells overexpressing *TOP2* (Fig. 8B). A physiologically relevant concentra-tion of 10 μM LS1, which alone caused less than ∼2-fold cell death in *TOP2* overexpressing cells, enhanced 1 or 5 μg/ml etoposide toxicity by 10 and 8-fold, respectively. We hypothesize that LS1 enhances etoposide toxicity by transiently stabilizing Top2ccs that serve as good targets for etoposide poisoning.

**Figure 9:**
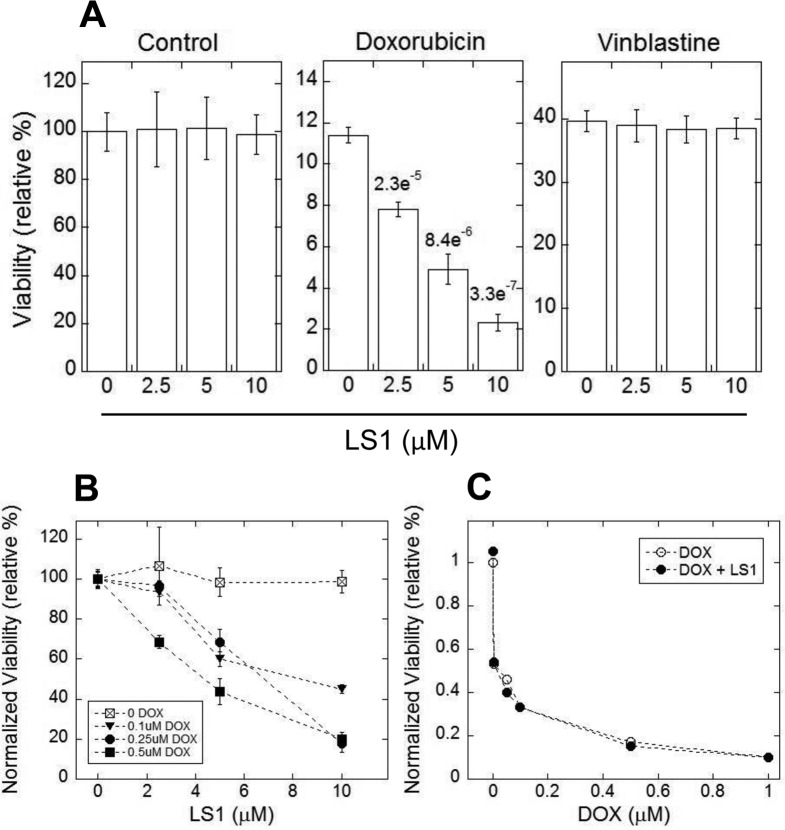
LS1 enhances doxorubicin killing of HT1080 fibrosarcoma cells (**A**) Cytotoxicity was measured in HT1080 cells for LS1 (left panel), DOX (middle panel), or vinblastine (right panel). (**B**) LS1 enhances killing of HT1080 cells at several concentrations of DOX. Experiments were performed as in (**A**). (**C**) Effect of LS1 on a primary human foreskin fibroblast cell line HCA2T that has been immortalized by transformation with the catalytic subunit of telomerase. HT1080 and HCA2T cells were seeded at low density. After 24 hours, cells were treated with DMSO (vehicle control) or DMSO containing DOX at the indicated concentrations without or with 10μM LS1. DMSO and DOX data were based on the average of 6 biological replicates. Vinblastine data was based on three biological replicates. Average values and standard deviation of the mean are plotted where available. P-values determined by student t-test are shown for DOX.

Based on the enhancement of etoposide cytotoxicity in yeast, we asked if LS1 might enhance the toxicity of Top2 poisons to human cancer cells. Doxorubicin is a frontline chemotherapeutic for both solid and liquid tumors. Enhancers have been sought because lifetime doses of doxorubicin are limited by cardiotoxicity [36, 37]. As a test of principle, human HT1080 fibrosarcoma cells were incubated with or without increasing concentrations of doxorubicin. As shown in Fig. 9A & B, LS1 alone is nontoxic to HT1080 cancer cells, but it enhances cell killing by doxorubicin up to fivefold. In contrast, LS1 did not enhance the toxicity of doxorubicin to noncancerous HCA2T human primary foreskin fibroblasts (Fig. 9C). LS1 also enhanced etoposide toxicity in HT1080 cells (data not shown). The fact that LS1 enhanced the toxicity of Top2 poisons in both yeast and human cancer cells indicates that it acts by poisoning Top2 in both species. The stimulatory interaction between LS1 and two chemically distinct Top2 poisons supports our conclusion that the physiological target of LS1 is Top2. We considered the possibility that LS1 might increase the potency of doxorubicin and etoposide by inhibiting multidrug resistance (MDR) pumps such as P-glycoprotein. Inhibition of P-glycoprotein increases intracellular concentrations of a broad range of xenobiotics, including vinblastine, resulting in greater drug potencies [38]. Since LS1 did not increase the toxicity of vinblastine in HT1080 cells (Fig. 9A, right panel) we conclude that the enhancement of doxorubicin toxicity by LS1 is not due to direct or indirect inhibition of MDR pumps.

### Reducing Top2 activity extends RLS

**Figure 10:**
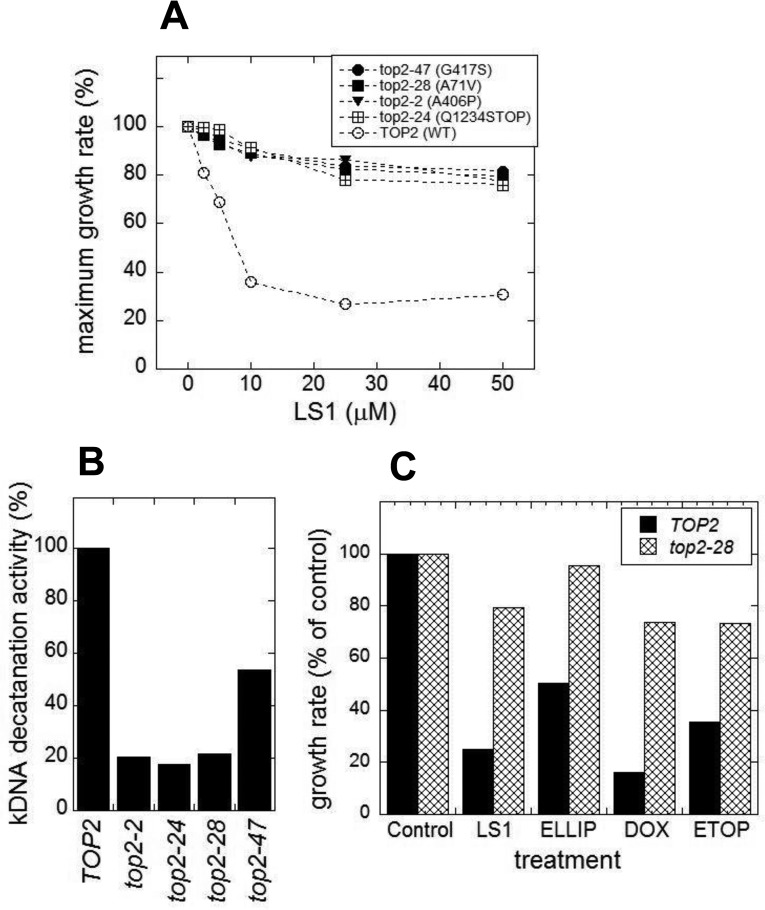
*top2* hypomorphic mutants confer resistance to LS1 and other Top2 poisons LS1 resistant *top2* mutants were selected as described in Methods and Methods using the host JN394 *top2ts2-4 rad52*Δ strain in combination with hydroxylamine mutagenesis of the YCpDED1-*TOP2* plasmid that complements the chromosomal *top2ts2-4* allele. (**A**) LS1 sensitivity of four representative LS1 resistant *top2* alleles. Growth rates were determined using the BioScreen C system and fit using the BGFit web-server as described in Materials and Methods. LS1 resistant *top2* alleles also confer resistance to other Top2 poisons. (**B**) Cell extracts from strains LS1 resistant *top2* alleles exhibit reduced Top2 activity using the kDNA assay. (**C**) *top2-28* cells exhibit increased resistance to multiple Top2 poisons, including LS1, ellipticine (ELLIP), etoposide (ETOP) and doxorubicin (DOX). All poisons were used at a concentration of 50μM and growth rates were compared to control (0.1% DMSO) and determined as in panel (**A**).

The above results are consistent with the hypothesis that LS1 enhances the potency of Top2 poisons in both yeast and human cancer cells by increasing the effective concentration of Top2 and it's intrinsic DNA damage-causing activity. This model suggests that reducing yTop2 activity could reduce intrinsic levels of DNA damage and actually extend RLS. Investigating the effects of reducing yTop2 levels must take into account that *TOP2* is an essential gene in yeast, and that both its' strong over- and under-expression cause growth defects [39, 40]. The first approach we took was to quantify DeaD lifespans of novel hypomorphic *top2* point mutants that were isolated by virtue of being resistant to LS1 (Fig. 10A), but exhibited no growth defects (not shown). These point mutations mapped to diverse sites within the protein (Fig. S6). Cell extracts from a number of these strains showed several-fold reduced yTop2 decatenation activities (Fig. 10B). As shown for one, *top2-28* (Fig. 10C), all of these mutants conferred resistance to doxorubicin, ellipticine and etoposide. The properties of these mutants are consistent with previously characterized hypomorphic alleles that, as would be expected from general loss of function mutations, confer resistance to multiple Top2 poisons [41–43].

**Figure 11:**
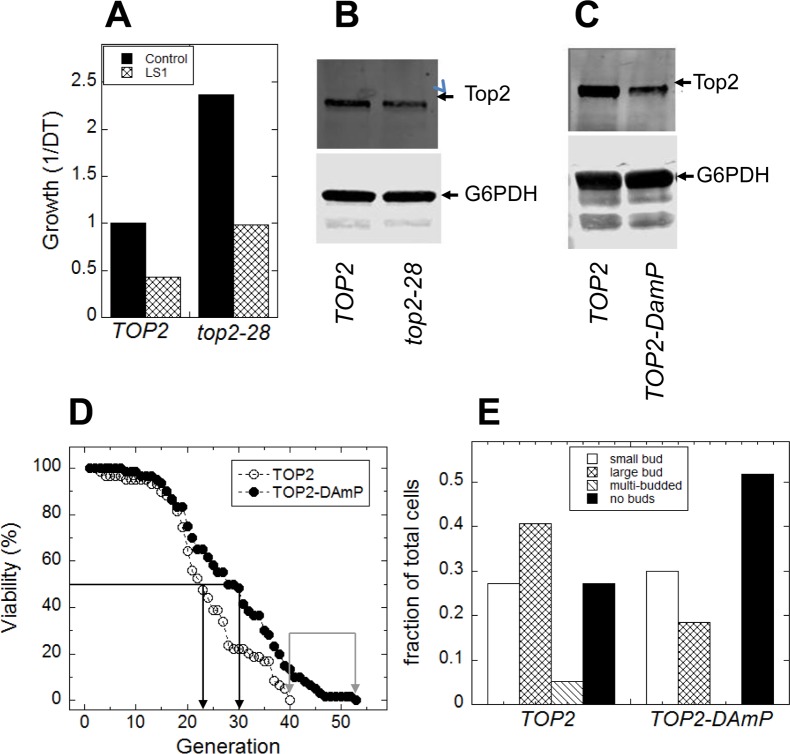
Reduced Top2 activity extends replicative lifespan (**A**) DeaD cells expressing *top2-28* displayed a roughly two-fold increase in lifespan both in the absence or presence of LS1 compared to cells expressing native *TOP2*. (**B**) Anti-Top2 immunoblot of cell extracts from *TOP2* and *top2-28* expressing DeaD strains, using anti-G6PDH as the loading control. (**C**) Anti-Top2 immunoblot of cell extracts from *Top2-DAmP* and TOP2 strains using anti-G6PDH as the loading control. (**D**) Microdissection RLS assays of isogenic haploid strains expressing either *Top2-DAmP* or *TOP2*, obtained by sporulation the heterodiploid. (**E**) Budding indexes of terminal mother cells taken from the microdissection RLS assays of *Top2-DamP* and *TOP2* strains shown in panel **C**.

As shown in Fig. 11A, *top2-28* cells, which express reduced yTop2 activity (Fig. 11B), exhibited a significantly longer DeaD lifespan when compared to cells expressing wild type *TOP2* (Fig. 11A). To validate and extend these DeaD assay results, we performed microdissection assays on a strain encoding an under-expressing *TOP2-DAmP* gene [44]. This strain expresses native *TOP2* at approximately 30% wild-type levels (Fig. 11C), but exhibited no apparent growth defect (not shown). Since the haploid strain containing the *TOP2-DAmP* allele was generated from the heterodiploid and compared to the haploid strain containing the *TOP2* wild-type allele, the strains were isogenic except at the *TOP2* locus. As shown in Fig.11D, constitutively reducing yTop2 levels extended both mean and maximum RLS.

The budding index of the terminally senescent mothers from the microdissection assays revealed that *TOP2*-*DAmP* cells tended to arrest more frequently as unbudded (G1) cells (Fig. 11E). Low budding indexes of senescent mother cell populations indicate robust growth control at G1/S checkpoints, and correlate with longer microdissection [28] and microfluidics-based RLS assays [45–47]. Therefore, wild type levels of yTop2, which are presumably tuned to optimize proliferative growth, are deleterious in aging cells—a phenomenon known as antagonistic pleiotropy [48, 49].

## DISCUSSION

We describe novel biological properties of a drug-like molecule identified in a high throughput DeaD assay screen for compounds that shorten yeast RLS. LS1 was originally described as the unsubstituted scaffold used in the development of less cytotoxic analogues of ellipticine [32]. In vivo and *in vitro* evidence show that LS1, like ellipticine, is a Top2 poison, albeit weaker and significantly less cytotoxic. LS1 exhibits none of the cytotoxicity to cancer (or normal) cells that ellipticine and other chemotherapeutic Top2 poisons show. LS1 inhibits the *in vitro* decatenation activity of human TOP2-α, and induces yTop2 to generate stable DSBs (Top2ccs) in plasmid DNA. Like known Top2 poisons, LS1 is toxic to cells overexpressing *TOP2* and to cells deleted for *RAD52* epistasis group genes. The latter synthetic interaction indicates that homologous recombination is needed to repair Top2ccs formed in the presence of LS1, and suggested the hypothesis that LS1 is toxic to aging cells, which, like young *rad52Δ* cells, may exhibit a diminished capacity to recognize and/or repair Top2ccs.

A key finding in our study is that yeast cells live longer when expressing reduced levels of yTop2. We propose that the Top2 poison activity of LS1 accelerates aging by presenting higher than normal Top2cc levels to aging cells with declining genome maintenance systems [14–17]. It follows that decreasing yTop2 levels would extend RLS by reducing DNA damage to levels below that which cause normal rates of senescence. The idea that Top2 is a natural source of DNA damage is not new. Top2-mediated DNA damage has previously been implicated in physiologically normal processes [11]. In mammals, TOP2β-induced DSBs are necessary for the regulated transcription of neuronal early-response genes [50] and for TOP2β mediated androgen-induced DNA rearrangements in prostate cancer [51]. *C. elegans top-2* is also responsible for a striking increase in DNA damage during zygotic genome activation [52]. The massive induction of transcription that accompanies entry of larval primordial germ cells into the cell cycle triggers widespread DNA damage and activates DNA damage checkpoints that slow entry into mitosis. Remarkably, reducing *top-2* expression both reduced levels of DSBs in germ line cells and accelerated their entry into mitosis, presumably by precluding the need for the DNA damage checkpoint. This phenomenon provides a precedent for our hypothesis that a reduction in yTop2 expression decreases DNA damage in old cells and extends RLS. As suggested by an apparent loss of checkpoint control and increased rates of loss of heterozygosity (LOH) in the daughters of old mothers [53, 54], old yeast cells proceed through mitosis before damaged chromosomes can be repaired.

Proving that a molecule selectively shortens RLS is challenging because even low levels of toxicity—either cytotoxic or cytostatic—will reduce apparent RLS by arresting cell division irrespective of age. An effective lifespan shortening agent should have little effect on the physiology of log-phase cells, ∼87% of which are 3 generations or younger, even when the target is an essential housekeeping factor like yTop2. As is the case with many drugs that target physiologically important targets, the dose-dependence of lifespan shortening compounds is critical to limiting the toxic side effects associated with targeting essential longevity factors. LS-1 does become toxic at very high doses, possibly due to over-inhibition of yTop2 or off-target effects, or a combination of both. The initial DeaD screen included both permissive and nonpermissive assays that provided a filter to exclude molecules such as doxorubicin and etoposide that are toxic under permissive conditions (Fig. 1C & D). In contrast, LS1 and NAM reduce DeaD lifespan at concentrations that have no apparent effects on permissive growth. Besides having no effect on growth rate, LS1 has no discernable effects on either the cell cycle or viability. The viability criterion is important because even low levels of nonage-associated cell death cause significant decreases in apparent RLS using the microdissection assay. For example, *rad52*Δ cells, which had previously been argued to be short-lived [28], died in log phase at a rate of ∼2%/generation. A 2% cell death rate has a negligible impact on proliferative growth, but reduces mean RLS by ∼30%. LS1 has no detectable effect on cell viability even at concentrations 70-fold higher than were sufficient to reduce RLS by ∼30%. We conclude that LS1 is a bona fide lifespan-shortening probe that is ∼200-fold more potent than NAM.

An age-associated decline in genome maintenance creates a situation where otherwise manageable levels of DNA damage become toxic. We propose that LS1 is selectively toxic to older cells that have lost the capacity to mitigate yTop2-induced DNA damage, either by failing to properly repair Top2ccs or by failing to efficiently activate the DNA damage checkpoint and stalling the cell cycle until the damage can be repaired. As described above, older diploid yeast mother cells switch to a state of high genome instability that continues to produce high levels of LOH in daughters until the mothers die [53, 54]. LOH in the daughters of older mother cells is primarily due to damage-prone breakage induced repair (BIR) of DSBs. Moreover, unlike young cells that delay the cell cycle in order to repair DSBs, old mothers that produce daughters with LOH do not exhibit cell cycle delay or arrest, consistent with a loss of the DNA damage checkpoint [53, 54]. These studies argue that LOH in aging yeast arises not from an increase in the rate of DNA damage, but rather in the loss of genome maintenance systems needed to repair damage. Increased genomic instability in the form of LOH also rises dramatically during chronolo-gical aging. Interestingly, although chronological and replicative lifespans of natural isolates are not correlated [4], variability among natural isolates in the lag between the rise in LOH and loss of viability during chronological aging does correlate with the RLS of natural isolates, and argues that the capacity to resist genome instability contributes to natural variation in RLS [55–57]. LS1 does not affect chronological lifespan (M. Weinberger and W. Burhans, personal communication), consistent with the notion that Top2 poisoning by LS1 is selectively toxic to replicating cells, presumably by interfering with genome maintenance during and following DNA replication.

Are our results consistent with what others have observed in aging yeast? Whereas Hu et al. [58] observed numerous genome rearrangements in older mother cells, Kaya et al. [55] sequenced the genomes of colonies produced by daughters of individual old mother cells and found only low numbers of mutations, effectively ruling out the accumulation of mutations in the mothers as the cause of senescence. Our results are consistent with both of these studies. A single unrepaired DSB in a mother cell that has lost the capacity to either sense or repair the damage can be lethal. This type of damage would not manifest itself as DNA damage in the genomes of daughter cells. Accordingly, in the case of yTop2-mediated DNA damage, it is not the accumulation of mutations that leads to senescence, but rather as few as a single catastrophic DSB—those not transmitted to daughters because the mothers die—that lead to age-associated cell death.

Our observation using microdissection RLS assays that terminal mothers under-expressing *TOP2* exhibit a higher frequency of arrest as unbudded G1/S cells compared to normal cells is consistent with improved checkpoint function. Consistent with the idea that normal levels of yTop2 cause RLS-limiting DNA damage and loss of growth control, Delaney et al. [28] reported that mutations that cause defects in genome stability genes tend to shorten RLS and increase the proportion of mothers that senesce as budded cells. Moreover, there is a strong statistical correlation between RLS and the budding index of terminal mothers, the latter of which can vary between ∼25-70%. Since not all mothers senesce as budded cells, it is unlikely that DNA damage and loss of growth control is the only mechanism that limits RLS in yeast. Thus we conclude only that yTop2-induced DNA damage contributes to aging.

Beyond studies in yeast, genome instability or defects in genome maintenance are strongly correlated with aging [2, 12, 59, 60]. Strong evidence in support of the DNA damage theory of aging includes progeroid syndromes in humans and rodents that are associated with mutations that affect genome maintenance (reviewed in [2]). Additional evidence comes from studies showing that DNA repair systems decline in older animals [61–63]. The specific role of DSBs in aging is supported by the appearance of age-associated phenotypes in mouse liver following the selective induction of DSBs in that tissue [64]. Age-associated defects in growth signaling, resulting in inappropriate entry into S-phase before DNA damage can be sensed and repaired, or before adequate stores of nucleotides are available, can lead to catastrophic events such as replication fork collapse and irreparable arrest at G2/M [65]. But these observations do not address whether DNA damage is cause or consequence of aging. What has been lacking in support of the DNA damage theory of aging are cases, such as we show in the case of yTop2, where lifespan is extended by reductions in DNA damage or by increases in repair systems or DNA damage checkpoints. Some evidence, such as the extended lifespan of male mice that overexpress SIRT6 [66], which is known to increase expression of DNA repair systems [67], are confounded by the pleiotropic roles of SIRT6 [68]. Similarly, RLS extension in yeast by overexpressing histones, which normally become depleted by about 50% in aging yeast, results in a global increase in transcription, suggests that this form of decline in genome maintenance plays a role in aging and, importantly, can be mitigated [69].

The antagonist pleiotropy (AP) theory of aging posits that the normal activities of some genes are beneficial during development and reproduction, but, for whatever reason(s), promote senescence later in life [48, 49]. The mutation accumulation (MA) theory posits that aging results from the accumulation of mutations that are selectively deleterious only late in life [48, 49]. Both theories depend on the assumption that AP genes or MA alleles escape natural selection because their phenotypes manifest post-reproductively. Our results indicate that yeast *TOP2* is an AP gene. If we assume that DNA damage by yTop2 occurs at approximately constant rates throughout yeast lifespan, then the AP properties of *TOP2* require an age-associated decline in genome maintenance similar to that which produces LOH in the daughters of old mother cells. This scenario is inconsistent with the MA theory of aging because most of these deleterious mutations would be deleterious whether they occurred early or late in life. In any case, Kaya et al. [55] did not find a significant accumulation of age-associated mutations. Thus our results with yTop2 support the AP theory of aging. yTop2 activity is required for proliferation in young cells, but it becomes toxic late in life. Thus yTop2 is indirectly anta-gonistically pleiotropic as its effect on longevity depends on an age-associated decline in genome maintenance. If correct, the AP effects of *TOP2* could be mitigated late in life, either by moderately reducing *TOP2* expression to levels that do not affect proliferation, which we have shown to be possible, or by enhancing repair or damage sensing and checkpoint systems later in life. In addition to yTop2, other DNA cleaving or modifying enzymes that have potential to cause DNA damage with AP properties such as Topoisomerase 1 [70, 71] may contribute to aging and age-associated diseases.

## MATERIALS AND METHODS

### Strains and LS1 chemical-genetic interactions

Negative chemical-genetic interactions with LS1 were initially determined by replica plating spots of similarly diluted mid-log cultures of the Yeast deletion collection library (GE Life Sciences; [72, 73]) on SCD and SCGal media lacking or containing 20μM LS1. Any patch showing less growth was confirmed more thoroughly by replica plating a series of spot dilutions of log-phase (OD600=1.0) cultures on media containing either DMSO (0.1%) in the absence or presence of various concentrations of LS1. To test if LS1 is a topoiso-merase poison, high copy number plasmids from the Yeast Tiling Collection (Thermo Scientific) containing *TOP1*, *TOP2*, and *TOP3* genes were isolated and transformed into the parental yeast strain BY4741 using Li-acetate/PEG with *LEU2* as the selectable marker. [74].

### Low to moderate throughput liquid culture DeaD assays

Strains carrying the three DeaD assay loci (*GAL1:CDC6*, *HO:CDC6*, and the partial deletion of the *ASH1* promoter) are grown two days on YP/2% galactose agar plates. A single colony of each strain is suspended in water and used to inoculate small (1-3 mL) cultures in SCRafGal (SC/ 1.9% raffinose/ 0.1% galactose, USBiologicals, Swampscott MA) for overnight growth to early log phase (to ensure early log phase by the next day, we typically do parallel inoculations at calculated optical densities (600nm) of 0.0001, 0.00003, and 0.00001). After overnight growth, one culture of each strain is used to inoculate SC/2% glucose and SCRaffGal at a calculated OD600 of 0.0001. Using a BioScreen C plate reader (Growth Curves USA, Piscataway NJ), cultures are grown at 30°C for 3 days, with an optical density (600 nm) reading taken every 20 minutes.

To calculate “DeaD lifespan,” we use the restrictive (i.e. SC/2% glucose) optical density reading at 60 hours, normalized to the estimated number of doublings that have occurred. To estimate the number of doublings, we use the actual number of doublings in permissive (i.e. SCRaffGal) medium in the first 24 hours, plus 36 times the calculated number of doublings per hour derived from the culture growth from hour 18 to hour 24. This assumes that the restrictive growth medium does not become limiting during the course of the assay, an assumption that is supported by restrictive optical density readings that are typically in the range of 0.1 to 0.3 at 60 hours. Growth rates expressed as the reciprocal of the doubling time (1/DT).

### *TOP2* mutagenesis and isolation of LS1-resistant yeast

A plasmid (YCpDED1-yTOP2) containing yeast *TOP2* gene under the control of DED1 promoter and strain JN394top2-4 (*MATa ura3-52 leu2 trp1 his7 ade1-2 ISE2 rad52::LEU2 top2-4*) containing a temperature sensitive allele of *TOP2* in a *rad52Δ* background were employed [26]. YCpDED1-yTop2 plasmid was maintained by URA selection. Hydroxylamine mutagenesis of the plasmid was performed as previously described [41, 75]. Mutations in the plasmid copy of the yeast TOP2 gene that retained function yet were resistant to LS1 were obtained by selection at the restrictive temperature (35°C) where the chromosomal *TOP2* ts2-4 allele is non-functional by including 20μM LS1 in the growth media. The resistance of individual strains carrying *TOP2* mutations was checked by plating spot-dilutions on SCD-URA plates containing either DMSO carrier or DMSO with 20μM LS1 added. To rule out potential mutations in the strains as a cause of LS1 resistance, strains were cured for the p*DED1*-y*TOP2* mutagenized plasmid by treatment with 5-floroorotic acid (5-FOA), and subsequently tested for LS1 resistance. Reciprocally, p*DED1*-y*TOP2* mutagenized plasmids were also rescued from strains showing resistance to LS1, transformed into naïve JN394t2-4 and re-tested for resistance to LS1. Growth in liquid media (SCD-URA) containing various concentrations of LS1 was measured in a Bioscreen C instrument (Growth Curves USA; Piscataway, NJ). Relative growth rates (ΔO.D.600/h) of JN394t2-4 containing p*DED1-yTOP2* (wild-type or mutant derivatives) were measured for up to 60h after an initial inoculum of .005 OD600 units. Growth data were fit to obtain maximum growth rates using the BGFit webserver [76].

### Inhibition of human Topoisomerase 2-alpha (hTop2-α)

LS1 inhibition of hTop2-α was determined by measuring the effect of LS1 on the ability of hTop2-α to decatenate kinetoplastid DNA from *Crithidia fasciculata*. Human Top2-α was added to 0.2μg of kDNA (both were purchased from Topogen, Inc; Buena Vista, CO) in the presence of 1% DMSO (vehicle control) without or with increasing concentrations of LS1 as indicated. Samples were incubated at 37°C for 15min, and then reactions were terminated by the addition of an SDS buffer. Samples were resolved by 1% agarose 1xTAE gel and stained with ethidium bromide. Decatenated versus intact kDNA species were quantified by densitometry using GelQuant.NET1.8.2 software. Final % products were plotted using KaleidaGraph software.

### Tissue culture cell growth

All cell lines were grown in monolayer at 37°C in 3% O^2^, 5% CO^2^ and 97% relative humidity in HERA Cell 240 incubators on treated polystyrene cell culture plates (Corning). Immortalized human fibroblast cells (HCA2T) were maintained in MEM (ATCC) supplemented with 15% FBS (Gibco) and 1x Pen/Strep (Gibco). Human fibrosarcoma cells (HT1080) were maintained in DMEM (Gibco) supplemented with 10% FBS (Gibco), 1x Pen/Strep (Gibco) and 1x nonessential amino acids (Gibco).

### Tissue culture cell survival

HT1080 and HCA2T cells were split to a density of 1 × 10^5^ cells per well of a six well plate 24 hours prior to treatment with the indicated concentration of DMSO (Sigma), Doxorubicin (Sigma), Vinblastine (Sigma) and LS1. All drugs were concurrently applied to the cells. Cell survival was measured 48 hours after treatment by counting the adherent cells in each group using a Z2 particle counter (Beckman Coulter). The ratio of adherent drug-treated cells to adherent cells treated with DMSO represents the raw survival. Experiments using doxorubicin were repeated six times; experiments using vinblastine were performed in triplicate.

### Western blotting

Anti-yTop2 polyclonal antibodies were purchased from Topogen (Buena Vista, CO). For the *TOP2* tiling experiments, protein extracts were prepared using the rapid boiling method [77]. When cells were grown in minimal media, protein extracts were prepared using the glass bead method [78]. Secondary antibodies conjugated to infrared dyes (LI-COR; Lincoln NE) were employed with an infrared gel scanner (LI-COR; Lincoln NE) for quantitation of the western blots.

### DNA intercalation assay

DNA Topoisomerase I (Top1) –based assay was used to assess the ability of LS-1 to intercalate. This approach relies upon linking number changes in supercoiled plasmid DNA induced by the intercalating molecule [79] which can be compared to DNA that was not treated. After treatment with the putative intercalator, linking number changes can be inferred after relaxation with DNA Top1. If intercalation occurred, upon removal of Top1, the final linking number (or state of supercoiling) will be distinct compared to DNA that was not treated (control). Molecules that fail to intercalate appear similar to control samples. DNA was incubated with DMSO alone or DMSO containing the indicated amount of doxorubicin, etoposide, or LS-1 for 15min at 25°C, followed by an additional 1h with added Top1. Next, DNA was extracted with phenol and chloroform and precipitated with ethanol. Samples were resolved by electrophoresis through 1% agarose 1xTAE gel and species were quantitated by densitometry with GelQuant.NET1.8.2 software.

## SUPPLEMENTARY MATERIAL


