# Supplementary material for: Effects of an unusual poison identify a lifespan role for Topoisomerase 2 in Saccharomyces cerevisiae
Source: Aging (Albany NY). 2017 Jan 5;9(1):68–87. doi: 10.18632/aging.101114 (PMC5310657; doi:10.18632/aging.101114)
Supplement: Supplementary file 1 [file aging-09-0068-s001.pdf]

## SUPPLEMENTARY MATERIAL

### Construction and characterization of an improved DeaD strain

K6001 is a W303-derived strain that was originally constructed for use in the study of mother cell-specific mating type switching [72, 73]. K6001 encodes two integrated copies of the essential gene *CDC6*, independently regulated by the repressible *GAL1* promoter and the mother cell-specific *HO* promoter, respectively (*GAL1:Ub:CDC6* and *HO:CDC6).* Previously, we exploited this strain as an alternative to the standard microdissection method of quantifying replicative lifespan (RLS), which is labor intensive and slow [74]. When K6001 cells are grown permissively in galactose-containing media (raffinose/galactose), *GAL1:CDC6* is expressed both in mother and daughter cells and the culture grows exponentially. When expression of the *GAL1:CDC6* gene is repressed by glucose, only the mother-cell-specific expression of *HO:CDC6* remains to support growth. Since *HO* expression is largely restricted to mother cells, daughters generally do not divide and growth of the culture is limited by the RLS of the initial cohort of mother cells. Since the success of this strategy depends on the conditional Death of Daughter cells, we call it the “DeaD” assay. Although promising, K6001 as a DeaD strain exhibited significant limitations. Its mother cells cease division on glucose after an average of only 3-4 divisions [74]. K6001 also has a short average lifespan under permissive conditions, presumably due to its W303 background (17 generations for K6001 [74], similar to 20.8 generations for W303R [18]). Early tests of the assay showed a reduction of DeaD lifespan by deletion of *SIR2* [74] but these tests ignored the fact that the *HO:CDC6* fusion is haploid-specific [75]. Deletion of *SIR2* prevents silencing of the mating type information at *HML* and *HMR* and thus renders cells pseudo-diploid [76, 77]. After a switch to glucose, *sir2Δ* mutants will thus be unable to express either copy of *CDC6* (see below for mechanism of HO gene expression) and will die rapidly, as was observed [74]. When the opposite mating type information was deleted from this strain, allowing *HO:CDC6* expression to sustain mother cells in glucose, no difference in DeaD lifespan was observed between *SIR2* and *sir2Δ* cells (data not shown). Combined with the other limitations, this finding indicated that the strategy exemplified by K6001 would have to be modified before it could be used as a platform for studying replicative lifespan.

We began by switching the parental background from W303 to the longer-lived S288C background [78]. For galactose-specific *CDC6* expression, we chose the strategy employed in K6001: a ubiquitin:*CDC6* fusion driven by the *GAL1* promoter. The N-terminal ubiquitin fusion allows modification of the amino terminus of Cdc6 to reduce protein stability and tighten control over Cdc6 activity. The ubiquitin moiety is co-translationally removed by ubiquitin processing proteases, and the amino acid serving as the new amino terminus of Cdc6 determines its half-life according to the N-end rule [79].

We cloned the *GAL:Ub:CDC6* fusion from K6001 to a plasmid vector. DNA sequencing revealed a tyrosine codon at the beginning of *CDC6*, rather than the expected arginine [73]. Multiple independent clones from two separately obtained K6001 isolates gave the same result. According to the N-end rule, tyrosine is less destabilizing than arginine [79]. Rapid turnover of *GAL1*-expressed Ub-Cdc6 is required for efficient death of daughters after a shift to glucose. Arginine, which is maximally destabilizing [79], is thus the desired N-terminal residue. All of the clones also carried a conservative mutation in the ubiquitin moiety: arginine 74 to lysine. We used PCR mutagenesis to correct this mutation and to change the N-terminal residue of Cdc6 to either arginine or methionine. We then integrated these alleles, and the original K6001 allele, in place of the endogenous *CDC6* in the S288C-derived strain Y7092 [80]. Since the *GAL:Ub:CDC6* allele is the only source of *CDC6* in these strains, all cells arrest when transferred to glucose, and the efficiency of this arrest is a function of the stability of Cdc6. Cells expressing arginine at the N-terminus of Cdc6 achieved the most efficient growth arrest. (Fig. S1A). This allele was incorporated into all subsequent DeaD strains.

The *HO:CDC6* allele in K6001 is an imprecise fusion of the open reading frame of *CDC6* to the *HO* promoter: the fusion leaves in place more than 90bp of the *CDC6* 5’ untranslated region, and all of the 3’ *CDC6* untranslated sequences. This construction excludes the 3’ UTR of *HO*, which appears to play a role in mother-cell-specificity of *HO* expression [81]. For our new DeaD strain, we therefore created a precise replacement of the *HO* open reading frame with *CDC6*, leaving the large *HO* promoter and the *HO* 3’ untranslated region in place. This *HO:CDC6:HO* fusion was integrated into a strain already carrying *GAL1:UbR:CDC6* to create DeaD strain BB573. BB573 mother cells have improved survival on glucose compared to K6001 mother cells: mean RLS increased from 3.4 to 8.3 generations ($p=5.94 \times 10^{-13}$, Fig. S1B). However, 8.3 generations is still much shorter than the 26-28 generation mean lifespan of normal SC288c-derived cells [78].

To further improve mother cell survival in glucose, we sought to increase mother cell expression of *HO:CDC6*. Insight into expression of this fusion can be gained from studies of normal *HO*. In cells that express the wild-type

HO endonuclease, mother cells switch mating type at a rate of ~70% [82]. Inhibition of switching in the remaining ~30% of mothers is dependent on the transcriptional repressor Ash1, since deletion of *ASH1* increases mother cell switching to 95-100% [72, 82]. These results indicate that Ash1 is normally incompletely excluded from mother cells. Ideally, Ash1 would be completely partitioned into the incipient daughter cell (the bud), leaving none in the mother cell to repress *HO* expression.

*ASH1* is expressed at the end of mitosis, when the transcription factors Swi5 and Ace2 enter the nucleus and promote ASH1 expression by binding to four putative Swi5/Ace2 binding sites (predicted by the nucleotide sequence kGCTGr, where "K" is G or T and "R" is A or G, [83] in the *ASH1* promoter. *ASH1* expression is dramatically decreased in *swi5 ace2* double mutants [72, 84]. We deleted the Swi5/Ace2 binding sites, either *in toto* or in pairwise combinations, in BB573 and assessed the effect on mother and daughter cell survival on glucose by pedigree analysis (Fig. S2). Deletion of the 1st two Swi5p binding sites (*ASH1-Δ12*) caused too drastic a drop in Ash1: daughter cell survival dramatically increased (represented by tall bars in Fig. S2 panel B), compare with parental strain BB573, Fig S2 panel A). This effect was caused to a lesser extent by deletion of the middle two sites (*ASH1-Δ23*, Fig. S2 panel C) or of all four binding sites (*ASH1-Δ14*, Fig S2 panel D). In contrast, deletion of the 3rd and 4th Swi5p binding sites produced a strain with enhanced mother cell survival without increased daughter cell "escape" (Fig S2 panel E). This strain, *ASH1-Δ34*, was renamed BB579 and it and its derivatives were used in all subsequent work. BB579 has a mean survival of 11.3 generations on glucose (longer than its parental strain, BB573, $p=1.77 \times 10^{-7}$) corresponding roughly to a model combining Gompertzian senescence with a stochastic death rate of 7.5% (Fig. S3 panel A).

Yeast cells become sterile near the end of their lifespan due to de-repression of the silent *HM* mating type loci and the resulting pseudo diploidy [85]. De-repression of the *HM* loci in a DeaD strain will result in failure to express haploid-specific *HO:CDC6*, as discussed above, and which might cause premature death in aging cells and an artificially short lifespan. To investigate the potential benefit of preventing pseudo diploidy by deleting one of the two mating type loci, *HMR*, we deleted HMR in *SIR2* and *sir2Δ* BB579 cells. We performed microdissection (Fig. S3 panels A and B) and DeaD lifespan assays (Fig. S3 panel C) of parental BB579 (*HMR*), *hmrΔ*, *sir2Δ* and *hmrΔ sirΔ* strains. Deletion of *SIR2* alone produced an extremely short apparent RLS, presumably due to repression of HO expression resulting from pseudo diploidy, and deletion of *HMR* and *SIR2* together gave an intermediate phenotype (Fig. S3 panels A and C) in both the microdissection and DeaD lifespan assays. None of these deletions had significant effects on permissive growth in galactose, although the *hmrΔ sir2Δ* strain exhibited a minor growth defect (Fig. S3 panel D). By microdissection RLS assay, deletion of *HMR* slightly extended RLS: mean RLS increased from 11.3 to 12.1 generations ($p=1.5 \times 10^{-2}$), and maximum increased from 32 to 45 (Fig. S3 panel B). Deletion of *SIR2* in *hmrΔ* BB579 cells shortened RLS to a mean of 8.6 generations ($p=1.45 \times 10^{-4}$), with a maximum of 33 generations (Fig. S3 panel A).

The above results demonstrate that *SIR2* strains have longer DeaD assay lifespans than *sir2Δ* strains in the improved BB579 background. To test this relationship further, and to determine whether the DeaD assay is sensitive not only to lifespan shortening but also to lifespan extension, we asked whether we could detect a range of effects on RLS by altering the expression level of *SIR2* with a series of promoter fusions. Replicative lifespan varies with *SIR2* expression level in yeast, since deletion of *SIR2* shortens lifespan and an extra copy of *SIR2* extends it [18]. There is likely to be an upper limit to lifespan extension by up-regulation of *SIR2*, however, since *SIR2* overexpression from the *GAL1* promoter causes toxicity and elevated rates of chromosome loss [86]. For our test, we used PCR-based integration to replace the endogenous *SIR2* promoter with the *CYC*, *ADH*, *TEF*, or *GPD* promoter [87] in an *HMR*-deleted BB579 derivative. DeaD assay lifespan of the resulting strains paralleled the predicted promoter strength (*CYC<ADH<TEF<GPD*, [87]), with strains carrying the strongest two promoters showing extension of DeaD assay lifespan (Fig. S4 panel C).

As expected if strong overexpression of *SIR2* is toxic, the *GPDpr:SIR2* fusion, which we expect to be more strongly expressed than the *TEFpr:SIR2* fusion [87], confers no additional advantage for lifespan (Fig. S4 panel C). None of these deletions had significant affects on permissive growth in galactose, although the *hmrΔ sir2Δ* strain exhibited a minor growth defect (Fig. S3 panel B).

In a parallel set of experiments, we replaced the endogenous *SIR2* promoter with the same promoter series in a wild-type S288C strain and measured replicative lifespan using the standard microdissection assay. The results (Fig.4S Panel D) are a striking parallel to the DeaD assay findings. The *TEF* promoter extended mean lifespan by a robust 45%, while the stronger *GPD* promoter showed a lesser extension of 22%. We conclude that improvements to K6001 engineered into BB579 allow recapitulation of key

features of yeast aging using high throughput capable liquid growth-based assays, including lifespan shortening and extension by under- and overexpression of *SIR2*.

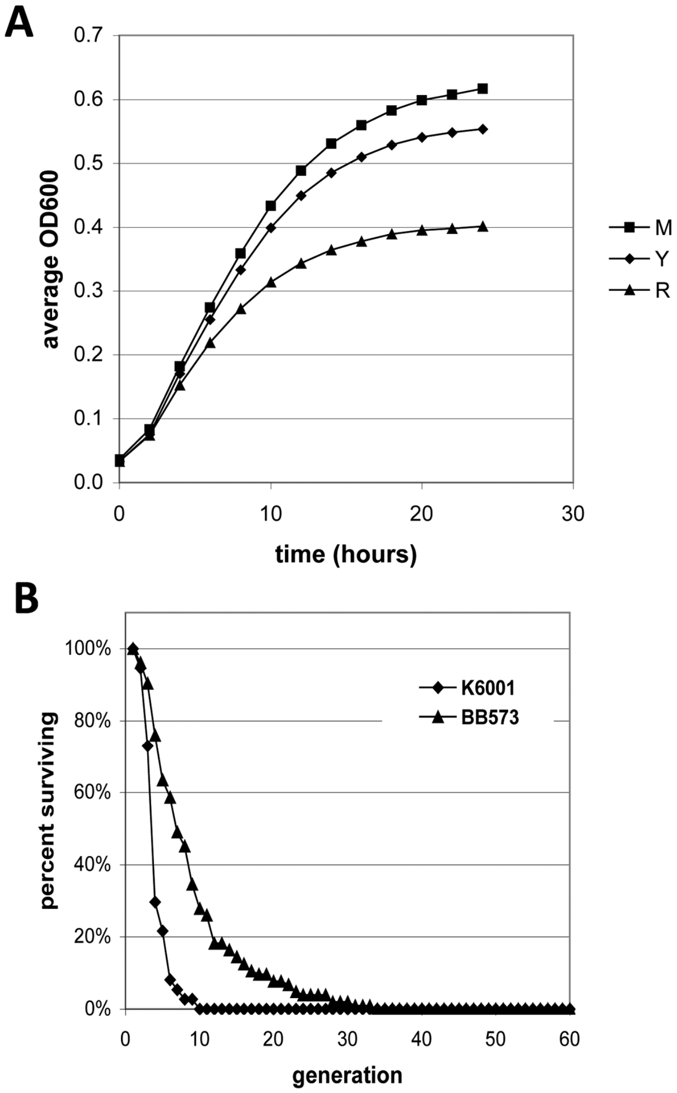


**Figure S1**. **Characterization of improved DeaD strain BB573.** (**A**) Arrest of *GAL:Ub:CDC6* strains in glucose is dependent on the N-terminal residue of Cdc6p. Cells were transferred to glucose at time 0 and growth arrest was monitored by measuring the culture OD600. The letter indicates the N-terminal Cdc6p residue: M: methionine, Y: tyrosine, or R: arginine. (**B**) BB573 mother cells have improved survival on glucose compared to K6001 mother cells: mean RLS increased from 3.4 to 8.3 generations ($p=5.94 \times 10^{-13}$).

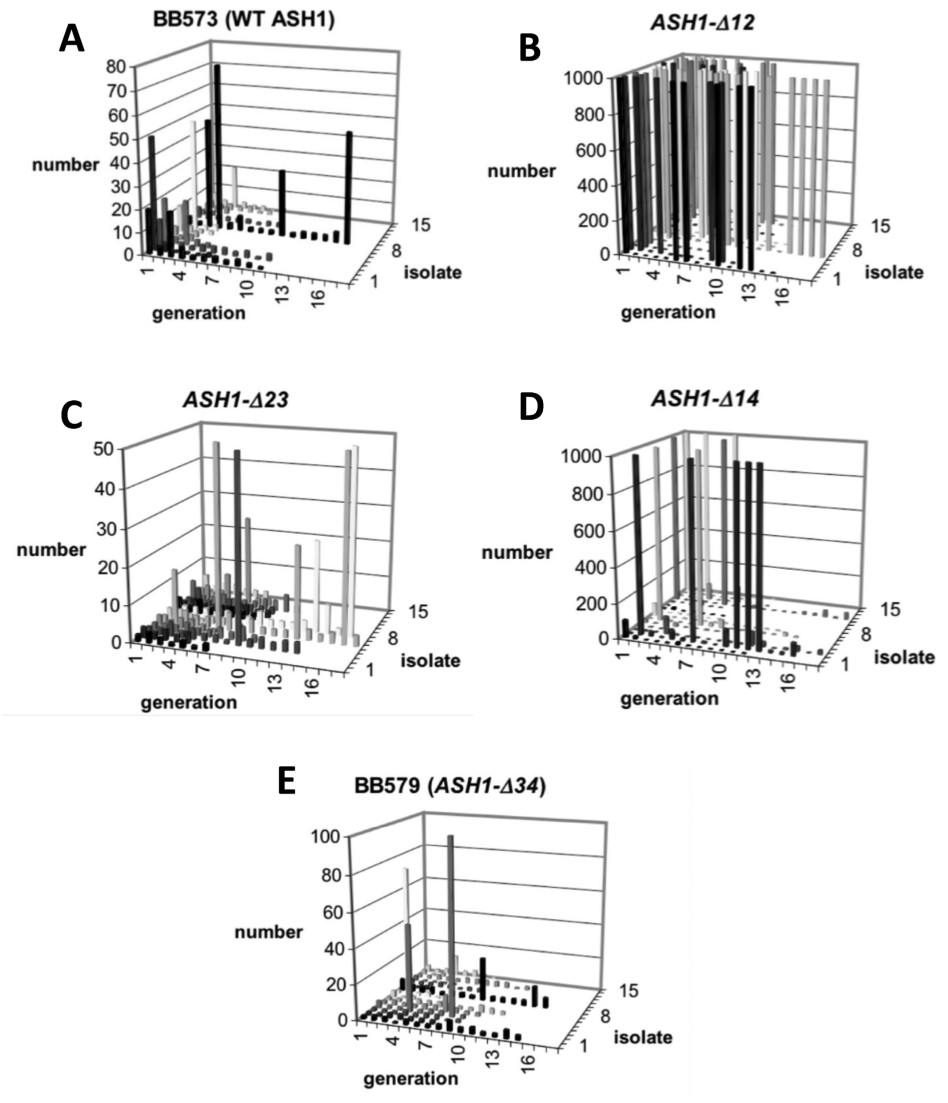


**Figure S2. Pedigree analysis of BB573 and derivatives carrying *ASH1* promoter deletions.** Strains were grown in a galactose-containing medium to mid-log phase and arrayed on glucose (SCD) plates for microdissection. Divisions of mother cells are represented on the x axis, and divisions of daughter cells by the height of the bars.

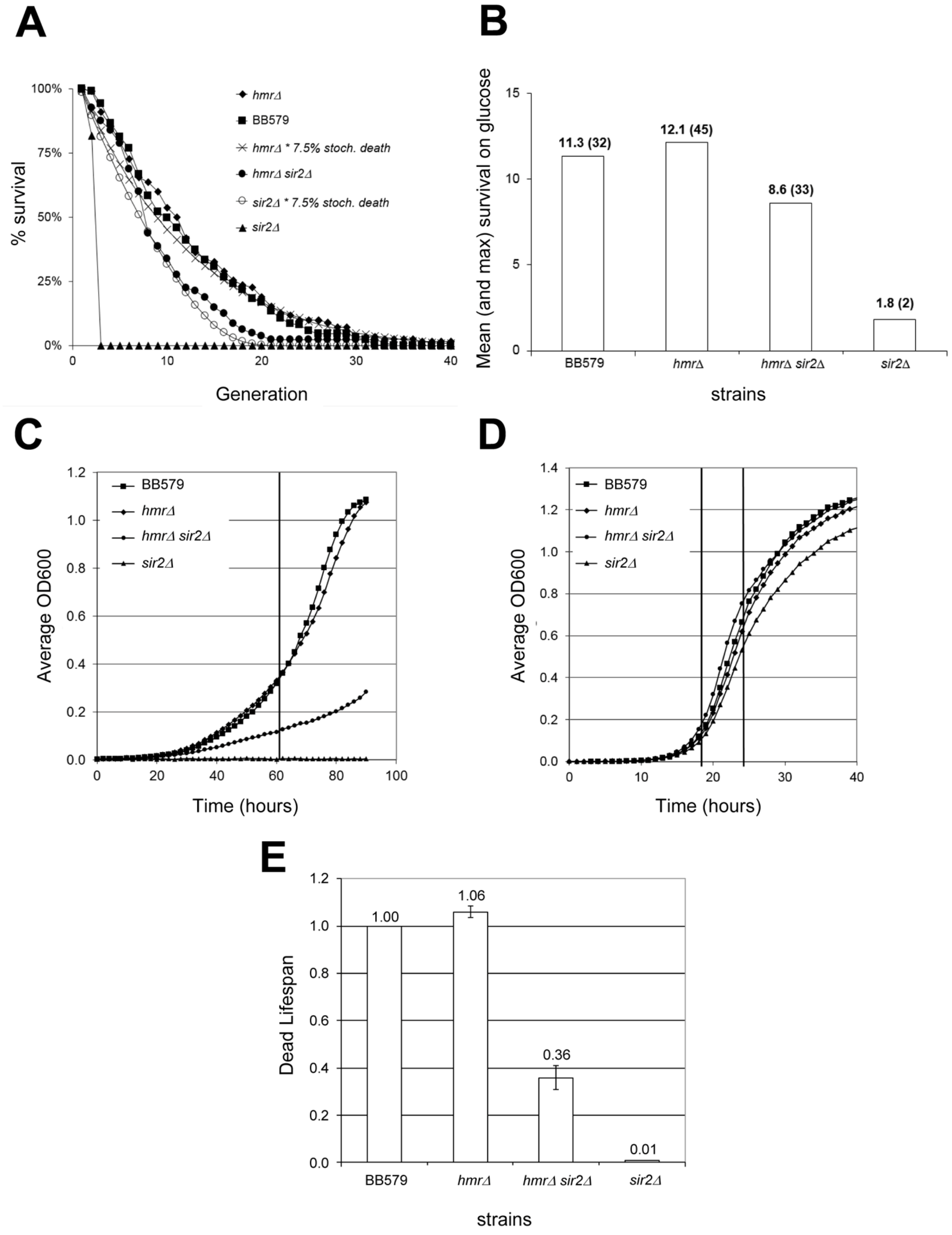


**Figure S3. The effect of *hmr* and *sir2* deletions on mother cell survival and DeaD lifespan in new DeaD assay strain BB579**. (**A**) Survival of BB579 and its *hmrΔ hmrΔsir2Δ*, and *sir2Δ* derivatives on glucose. Strains were grown to mid-log phase in a galactose-containing medium and arrayed on glucose (SCD) plates. Mother cell survival was determined by microdissection. For comparison, models of BY4741 ("x"s) and its *sir2Δ* derivative (open circles) with a 7.5% rate of stochastic death are included. (**B**) Mean and maximum replicative lifespans from (**A**). (**C**) Representative restrictive liquid cultures of the same four strains. (**D**) Parallel permissive liquid cultures. (**E**) Relative DeaD assay lifespans of the four strains, using data from (**C**) and (**D**) together with two more independent experiments. Error bars are +/- one standard error of the mean of the three experiments.

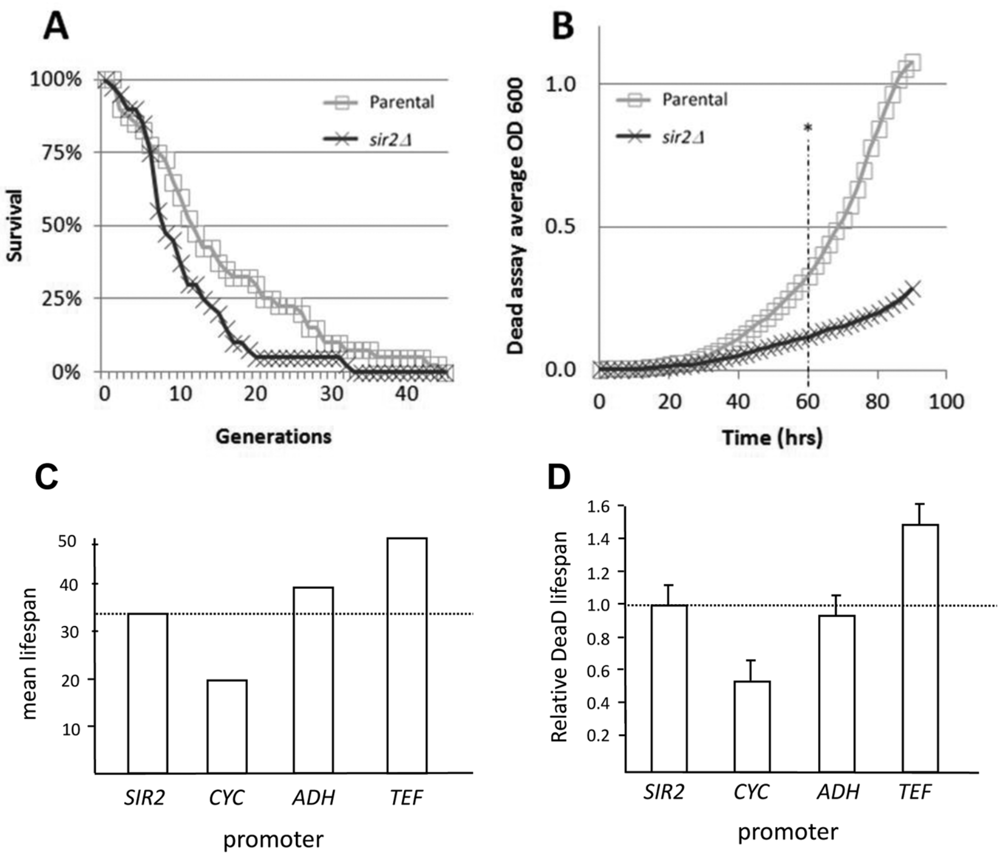


**Figure S4. DeaD assay recapitulates microdissection replicative lifespan measurement.** (**A**) In the traditional microdissection replicative lifespan method the DEAD strain deletion of *SIR2* results in a drop in average lifespan from 15.5 generations (□ Parental) to 10.5 generation (X *sir2Δ*), a 32.25% decrease. (**B**) In the DeaD assay the average lifespan decrease (* measured at 60hrs) from optical density of 0.331 (□ Parental) to 0.116 (X *sir2Δ*), a 65% drop in lifespan. (**C**) & (**D**) When *SIR2* expression levels are varied by promoters of varying strength from low (CYC), normal (ADH) and high (TEF), relative and mean lifespan as reported by the DeaD assay also show reduced, normal, and extended levels.

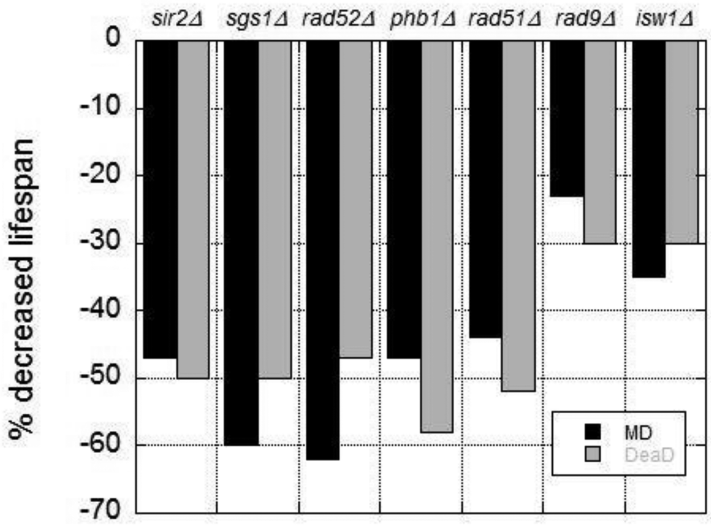


**Figure S5. DeaD assay reports replicative lifespan for several strains that show reduced lifespan in the traditional microdissection assay.** Yeast strains were obtained from the gene deletion collection. Replicative lifespan (%) for both microdissection and DeaD assay were determined as described in Methods.

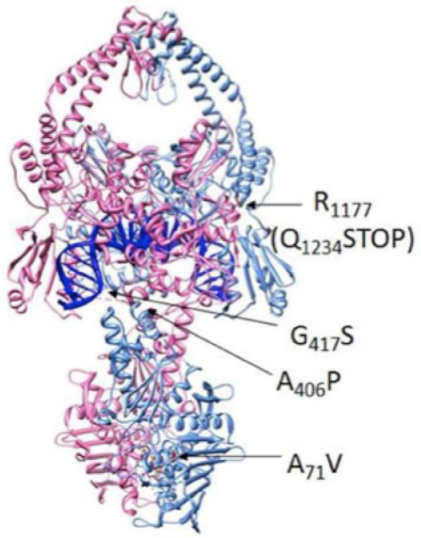


**Figure S6. Map of LS1 resistant hypomorphic *top2* alleles.** UCSF Chimera was used to display the X-ray structure of yeast Top2 and map the location of each of the LS1 resistant hypomorphic alleles onto the protein structure. The dispersed locations of each mutation are consistent with other *top2* alleles previously found to confer broad resistance to Top2 poisons such as DOX or ETOP.

## SUPPLEMENTARY REFERENCES


1. Bitto A, Wang AM, Bennett CF and Kaeberlein M. Biochemical Genetic Pathways that Modulate Aging in Multiple Species. Cold Spring Harb Perspect Med. 2015; 5:11.
2. Vermeij WP, Hoeijmakers JH and Pothof J. Genome Integrity in Aging: Human Syndromes, Mouse Models, and Therapeutic Options. Annu Rev Pharmacol Toxicol. 2016; 56:427-445.
3. Longo VD, Shadel GS, Kaeberlein M and Kennedy B. Replicative and chronological aging in Saccharomyces cerevisiae. Cell Metab. 2012; 16:18-31.
4. Qin H and Lu M. Natural variation in replicative and chronological life spans of Saccharomyces cerevisiae. Exp Gerontol. 2006; 41:448-456.
5. Lindstrom DL and Gottschling DE. The mother enrichment program: a genetic system for facile replicative life span analysis in Saccharomyces cerevisiae. Genetics. 2009; 183:413-422, 411SI-413SI.
6. Zhang Y, Luo C, Zou K, Xie Z, Brandman O, Ouyang Q and Li H. Single cell analysis of yeast replicative aging using a new generation of microfluidic device. PLoS One. 2012; 7:e48275.
7. Pommier Y, Leo E, Zhang H and Marchand C. DNA topoisomerases and their poisoning by anticancer and antibacterial drugs. Chem Biol. 2010; 17:421-433.
8. Nitiss JL. Targeting DNA topoisomerase II in cancer chemotherapy. Nat Rev Cancer. 2009; 9:338-350.
9. Deweese JE and Osheroff N. The DNA cleavage reaction of topoisomerase II: wolf in sheep's clothing. Nucleic Acids Res. 2009; 37:738-748.
10. Vijg J and Suh Y. Genome instability and aging. Annu Rev Physiol. 2013; 75:645-668.
11. Cortes Ledesma F, El Khamisy SF, Zuma MC, Osborn K and Caldecott KW. A human 5'-tyrosyl DNA phosphodiesterase that repairs topoisomerase-mediated DNA damage. Nature. 2009; 461:674-678.
12. Nitiss JL and Nitiss KC. Tdp2: a means to fixing the ends. PLoS Genet. 2013; 9:e1003370.
13. Pommier Y, Huang SY, Gao R, Das BB, Murai J and Marchand C. Tyrosyl-DNA-phosphodiesterases (TDP1 and TDP2). DNA Repair (Amst). 2014; 19:114-129.
14. Malik M, Nitiss KC, Enriquez-Rios V and Nitiss JL. Roles of nonhomologous end-joining pathways in surviving topoisomerase II-mediated DNA damage. Mol Cancer Ther. 2006; 5:1405-1414.
15. Sabourin M, Nitiss JL, Nitiss KC, Tatebayashi K, Ikeda H and Osheroff N. Yeast recombination pathways triggered by topoisomerase II-mediated DNA breaks. Nucleic Acids Res. 2003; 31:4373-4384.
16. Sinclair DA and Guarente L. Extrachromosomal rDNA circles--a cause of aging in yeast. Cell. 1997; 91:1033-1042.
17. Defossez PA, Prusty R, Kaeberlein M, Lin SJ, Ferrigno P, Silver PA, Keil RL and Guarente L. Elimination of replication block protein Fob1 extends the life span of yeast mother cells. Mol Cell. 1999; 3:447-455.
18. Kaeberlein M, McVey M and Guarente L. The SIR2/3/4 complex and SIR2 alone promote longevity in Saccharomyces cerevisiae by two different mechanisms.

Genes Dev. 1999; 13:2570-2580.

19. Tsuchiya M, Dang N, Kerr EO, Hu D, Steffen KK, Oakes JA, Kennedy BK and Kaeberlein M. Sirtuin-independent effects of nicotinamide on lifespan extension from calorie restriction in yeast. Aging Cell. 2006; 5:505-514.

20. Goto T and Wang JC. Cloning of yeast TOP1, the gene encoding DNA topoisomerase I, and construction of mutants defective in both DNA topoisomerase I and DNA topoisomerase II. Proc Natl Acad Sci U S A. 1985; 82:7178-7182.

21. Delaney JR, Chou A, Olsen B, Carr D, Murakami C, Ahmed U, Sim S, An EH, Castanza AS, Fletcher M, Higgins S, Holmberg M, Hui J, et al. End-of-life cell cycle arrest contributes to stochasticity of yeast replicative aging. FEMS Yeast Res. 2013; 13:267-276.

22. Powers RW, 3rd, Kaeberlein M, Caldwell SD, Kennedy BK and Fields S. Extension of chronological life span in yeast by decreased TOR pathway signaling. Genes Dev. 2006; 20:174-184.

23. Gorbunova V, Seluanov A, Mao Z and Hine C. Changes in DNA repair during aging. Nucleic Acids Res. 2007; 35:7466-7474.

24. Tewey KM, Chen GL, Nelson EM and Liu LF. Intercalative antitumor drugs interfere with the breakage-reunion reaction of mammalian DNA topoisomerase II. J Biol Chem. 1984; 259:9182-9187.

25. Wilhelmsson LM, Kingi N and Bergman J. Interactions of antiviral indolo[2,3-b]quinoxaline derivatives with DNA. J Med Chem. 2008; 51(24):7744-7750.

26. Nitiss JL, Liu YX, Harbury P, Jannatipour M, Wasserman R and Wang JC. Amsacrine and etoposide hypersensitivity of yeast cells overexpressing DNA topoisomerase II. Cancer Res. 1992; 52:4467-4472.

27. Arimondo PB, Baldeyrou B, Laine W, Bal C, Alphonse FA, Routier S, Coudert G, Merour JY, Colson P, Houssier C and Bailly C. DNA interaction and cytotoxicity of a new series of indolo[2,3-b]quinoxaline and pyridopyrazino[2,3-b]indole derivatives. Chem Biol Interact. 2001; 138:59-75.

28. Jensen LH, Nitiss KC, Rose A, Dong J, Zhou J, Hu T, Osheroff N, Jensen PB, Sehested M and Nitiss JL. A novel mechanism of cell killing by anti-topoisomerase II bisdioxopiperazines. J Biol Chem. 2000; 275:2137-2146.

29. Xiao H, Mao Y, Desai SD, Zhou N, Ting CY, Hwang J and Liu LF. The topoisomerase IIbeta circular clamp arrests transcription and signals a 26S proteasome pathway. Proc Natl Acad Sci U S A. 2003; 100:3239-3244.

30. Aryal B, Jeong J and Rao VA. Doxorubicin-induced carbonylation and degradation of cardiac myosin binding protein C promote cardiotoxicity. Proc Natl Acad Sci U S A. 2014; 111:2011-2016.

31. Lotrionte M, Biondi-Zoccai G, Abbate A, Lanzetta G, D'Ascenzo F, Malavasi V, Peruzzi M, Frati G and Palazzoni G. Review and meta-analysis of incidence and clinical predictors of anthracycline cardiotoxicity. Am J Cardiol. 2013; 112:1980-1984.

32. Sauna ZE, Kim IW and Ambudkar SV. Genomics and the mechanism of P-glycoprotein (ABCB1). J Bioenerg Biomembr. 2007; 39:481-487.

33. Holm C, Goto T, Wang JC and Botstein D. DNA topoisomerase II is required at the time of mitosis in yeast. Cell. 1985; 41:553-563.

34. Worland ST and Wang JC. Inducible overexpression, purification, and active site mapping of DNA topoisomerase II from the yeast Saccharomyces cerevisiae. J Biol Chem. 1989; 264:4412-4416.

35. Patel S, Sprung AU, Keller BA, Heaton VJ and Fisher LM. Identification of yeast DNA topoisomerase II mutants resistant to the antitumor drug doxorubicin: implications for the mechanisms of doxorubicin action and cytotoxicity. Mol Pharmacol. 1997; 52:658-666.

36. Jiang X. Random mutagenesis of the B'A' core domain of yeast DNA topoisomerase II and large-scale screens of mutants resistant to the anticancer drug etoposide. Biochem Biophys Res Commun. 2005; 327:597-603.

37. Vassetzky YS, Alghisi GC and Gasser SM. DNA topoisomerase II mutations and resistance to anti-tumor drugs. Bioessays. 1995; 17:767-774.

38. Breslow DK, Cameron DM, Collins SR, Schuldiner M, Stewart-Ornstein J, Newman HW, Braun S, Madhani HD, Krogan NJ and Weissman JS. A comprehensive strategy enabling high-resolution functional analysis of the yeast genome. Nat Methods. 2008; 5:711-718.

39. Jo MC, Liu W, Gu L, Dang W and Qin L. High-throughput analysis of yeast replicative aging using a microfluidic system. Proc Natl Acad Sci U S A. 2015; 112:9364-9369.

40. Lee SS, Avalos Vizcarra I, Huberts DH, Lee LP and Heinemann M. Whole lifespan microscopic observation of budding yeast aging through a microfluidic dissection platform. Proc Natl Acad Sci U S A. 2012; 109:4916-4920.

41. Liu P, Young TZ and Acar M. Yeast Replicator: A High-Throughput Multiplexed Microfluidics Platform for Automated Measurements of Single-Cell Aging. Cell Rep. 2015; 13:634-644.

42. Ljubuncic P and Reznick AZ. The evolutionary theories of aging revisited--a mini-review. Gerontology. 2009;

55:205-216.

43. Vijg J. Aging genomes: a necessary evil in the logic of life. Bioessays. 2014; 36:282-292.

44. Haffner MC, Aryee MJ, Toubaji A, Esopi DM, Albadine R, Gurel B, Isaacs WB, Bova GS, Liu W, Xu J, Meeker AK, Netto G, De Marzo AM, et al. Androgen-induced TOP2B-mediated double-strand breaks and prostate cancer gene rearrangements. Nat Genet. 2010; 42:668-675.

45. Butuci M, Williams AB, Wong MM, Kramer B and Michael WM. Zygotic Genome Activation Triggers Chromosome Damage and Checkpoint Signaling in C. elegans Primordial Germ Cells. Dev Cell. 2015; 34:85-95.

46. Haber JE. Aging: the sins of the parents. Curr Biol. 2003; 13:R843-845.

47. McMurray MA and Gottschling DE. An age-induced switch to a hyper-recombinational state. Science. 2003; 301:1908-1911.

48. Kaya A, Lobanov AV and Gladyshev VN. Evidence that mutation accumulation does not cause aging in Saccharomyces cerevisiae. Aging Cell. 2015; 14:366-371.

49. Kaya A, Ma S, Wasko B, Lee M, Kaeberlein M and Gladyshev VN. Defining Molecular Basis for Longevity Traits in Natural Yeast Isolates. NPJ Aging Mech Dis. 2015; 1.

50. Qin H, Lu M and Goldfarb DS. Genomic instability is associated with natural life span variation in Saccharomyces cerevisiae. PLoS One. 2008; 3:e2670.

51. Moskalev AA, Shaposhnikov MV, Plyusnina EN, Zhavoronkov A, Budovsky A, Yanai H and Fraifeld VE. The role of DNA damage and repair in aging through the prism of Koch-like criteria. Ageing Res Rev. 2013; 12:661-684.

52. Hart RW and Setlow RB. Correlation between deoxyribonucleic acid excision-repair and life-span in a number of mammalian species. Proc Natl Acad Sci U S A. 1974; 71:2169-2173.

53. MacRae SL, Croken MM, Calder RB, Aliper A, Milholland B, White RR, Zhavoronkov A, Gladyshev VN, Seluanov A, Gorbunova V, Zhang ZD and Vijg J. DNA repair in species with extreme lifespan differences. Aging (Albany NY). 2015; 7:1171-1184.

54. MacRae SL, Zhang Q, Lemetre C, Seim I, Calder RB, Hoeijmakers J, Suh Y, Gladyshev VN, Seluanov A, Gorbunova V, Vijg J and Zhang ZD. Comparative analysis of genome maintenance genes in naked mole rat, mouse, and human. Aging Cell. 2015; 14:288-291.

55. Burhans WC and Weinberger M. DNA damage and DNA replication stress in yeast models of aging. Subcell Biochem. 2012; 57:187-206.

56. Zeng Z, Cortes-Ledesma F, El Khamisy SF and Caldecott KW. TDP2/TTRAP is the major 5'-tyrosyl DNA phosphodiesterase activity in vertebrate cells and is critical for cellular resistance to topoisomerase II-induced DNA damage. J Biol Chem. 2011; 286:403-409.

57. Zeng Z, Sharma A, Ju L, Murai J, Umans L, Vermeire L, Pommier Y, Takeda S, Huylebroeck D, Caldecott KW and El-Khamisy SF. TDP2 promotes repair of topoisomerase I-mediated DNA damage in the absence of TDP1. Nucleic Acids Res. 2012; 40:8371-8380.

58. Alvarez-Quilon A, Serrano-Benitez A, Lieberman JA, Quintero C, Sanchez-Gutierrez D, Escudero LM and Cortes-Ledesma F. ATM specifically mediates repair of double-strand breaks with blocked DNA ends. Nat Commun. 2014; 5:3347.

59. Furniss KL, Tsai HJ, Byl JA, Lane AB, Vas AC, Hsu WS, Osheroff N and Clarke DJ. Direct monitoring of the strand passage reaction of DNA topoisomerase II triggers checkpoint activation. PLoS Genet. 2013; 9:e1003832.

60. Kanfi Y, Naiman S, Amir G, Peshti V, Zinman G, Nahum L, Bar-Joseph Z and Cohen HY. The sirtuin SIRT6 regulates lifespan in male mice. Nature. 2012; 483:218-221.

61. Mao Z, Tian X, Van Meter M, Ke Z, Gorbunova V and Seluanov A. Sirtuin 6 (SIRT6) rescues the decline of homologous recombination repair during replicative senescence. Proc Natl Acad Sci U S A. 2012; 109:11800-11805.

62. Kugel S and Mostoslavsky R. Chromatin and beyond: the multitasking roles for SIRT6. Trends Biochem Sci. 2014; 39:72-81.

63. Feser J, Truong D, Das C, Carson JJ, Kieft J, Harkness T and Tyler JK. Elevated histone expression promotes life span extension. Mol Cell. 2010; 39:724-735.

64. Giaever G, Chu AM, Ni L, Connelly C, Riles L, Veronneau S, Dow S, Lucau-Danila A, Anderson K, Andre B, Arkin AP, Astromoff A, El-Bakkoury M, et al. Functional profiling of the Saccharomyces cerevisiae genome. Nature. 2002; 418:387-391.

65. Winzeler EA, Shoemaker DD, Astromoff A, Liang H, Anderson K, Andre B, Bangham R, Benito R, Boeke JD, Bussey H, Chu AM, Connelly C, Davis K, et al. Functional characterization of the S. cerevisiae genome by gene deletion and parallel analysis. Science. 1999; 285:901-906.

66. Jones GM, Stalker J, Humphray S, West A, Cox T, Rogers J, Dunham I and Prelich G. A systematic library for comprehensive overexpression screens in Saccharomyces cerevisiae. Nat Methods. 2008; 5:239-241.

67. Sikorski RS and Boeke JD. In vitro mutagenesis and plasmid shuffling: from cloned gene to mutant yeast. Methods Enzymol. 1991; 194:302-318.

68. Verissimo A, Paixao L, Neves AR and Vinga S. BGFit: management and automated fitting of biological growth curves. BMC Bioinformatics. 2013; 14:283.

69. Horvath A and Riezman H. Rapid protein extraction from Saccharomyces cerevisiae. Yeast. 1994; 10:1305-1310.

70. Conzelmann A, Riezman H, Desponds C and Bron C. A major 125-kd membrane glycoprotein of Saccharomyces cerevisiae is attached to the lipid bilayer through an inositol-containing phospholipid. EMBO J. 1988; 7:2233-2240.

71. Waring M. Variation of the supercoils in closed circular DNA by binding of antibiotics and drugs: evidence for molecular models involving intercalation. J Mol Biol. 1970; 54:247-279.

72. Bobola N, Jansen RP, Shin TH and Nasmyth K. Asymmetric accumulation of Ash1p in postanaphase nuclei depends on a myosin and restricts yeast mating-type switching to mother cells. Cell. 1996; 84:699-709.

73. Piatti S, Bohm T, Cocker JH, Diffley JF and Nasmyth K. Activation of S-phase-promoting CDKs in late G1 defines a "point of no return" after which Cdc6 synthesis cannot promote DNA replication in yeast. Genes Dev. 1996; 10:1516-1531.

74. Jarolim S, Millen J, Heeren G, Laun P, Goldfarb DS and Breitenbach M. A novel assay for replicative lifespan in Saccharomyces cerevisiae. FEMS Yeast Res. 2004; 5:169-177.

75. Nasmyth K and Shore D. Transcriptional regulation in the yeast life cycle. Science. 1987; 237:1162-1170.

76. Rine J and Herskowitz I. Four genes responsible for a position effect on expression from HML and HMR in Saccharomyces cerevisiae. Genetics. 1987; 116:9-22.

77. Shore D, Squire M and Nasmyth KA. Characterization of two genes required for the position-effect control of yeast mating-type genes. Embo J. 1984; 3:2817-2823.

78. Kaeberlein M, Kirkland KT, Fields S and Kennedy BK. Genes determining yeast replicative life span in a long-lived genetic background. Mech Ageing Dev. 2005; 126:491-504.

79. Bachmair A, Finley D and Varshavsky A. In vivo half-life of a protein is a function of its amino-terminal residue. Science. 1986; 234:179-186.

80. Tong AH, Evangelista M, Parsons AB, Xu H, Bader GD, Page N, Robinson M, Raghibizadeh S, Hogue CW, Bussey H, Andrews B, Tyers M and Boone C. Systematic genetic analysis with ordered arrays of yeast deletion mutants. Science. 2001; 294:2364-2368.

81. Tadauchi T, Matsumoto K, Herskowitz I and Irie K. Post-transcriptional regulation through the HO 3'-UTR by Mpt5, a yeast homolog of Pumilio and FBF. EMBO J. 2001; 20:552-561.

82. Sil A and Herskowitz I. Identification of asymmetrically localized determinant, Ash1p, required for lineage-specific transcription of the yeast HO gene. Cell. 1996; 84:711-722.

83. Harbison CT, Gordon DB, Lee TI, Rinaldi NJ, Macisaac KD, Danford TW, Hannett NM, Tagne JB, Reynolds DB, Yoo J, Jennings EG, Zeitlinger J, Pokholok DK, et al. Transcriptional regulatory code of a eukaryotic genome. Nature. 2004; 431:99-104.

84. McBride HJ, Yu Y and Stillman DJ. Distinct regions of the Swi5 and Ace2 transcription factors are required for specific gene activation. J Biol Chem. 1999; 274:21029-21036.

85. Smeal T, Claus J, Kennedy B, Cole F and Guarente L. Loss of transcriptional silencing causes sterility in old mother cells of S. cerevisiae. Cell. 1996; 84:633-642.

86. Holmes SG, Rose AB, Steuerle K, Saez E, Sayegh S, Lee YM and Broach JR. Hyperactivation of the silencing proteins, Sir2p and Sir3p, causes chromosome loss. Genetics. 1997; 145:605-614.

87. Janke C, Magiera MM, Rathfelder N, Taxis C, Reber S, Maekawa H, Moreno-Borchart A, Doenges G, Schwob E, Schiebel E and Knop M. A versatile toolbox for PCR-based tagging of yeast genes: new fluorescent proteins, more markers and promoter substitution cassettes. Yeast. 2004; 21:947-962.
